# Supplementary material for: Prevalence and determinants of unintended pregnancy in Ethiopia: A systematic review and meta-analysis of observational studies
Source: PLoS One. 2020 Apr 7;15(4):e0231012. doi: 10.1371/journal.pone.0231012 (PMC7138300; doi:10.1371/journal.pone.0231012)
Supplement: S1 File — (DOCX) [file pone.0231012.s002.docx]

**Additional file 1. List of excluded references and reasons for exclusion.**

| **References** | **Reasons** |
| --- | --- |
| 1. Gessessew A. Abortion and unwanted pregnancy in Adigrat zonal hospital, Tigray, north Ethiopia. African journal of reproductive health. 2010;14(3):183-8. | 3 |
| 1. Ali A, Ali SA, Aziz Ali S, Khuwaja NS. Determinants of Unintended Pregnancy among Women of Reproductive Age in Developing Countries: A Narrative Review. Journal of Midwifery and Reproductive Health. 2016;4(1):513-21. | 1 |
| 1. Singh A, Singh A, Mahapatra B. The consequences of unintended pregnancy for maternal and child health in rural India: evidence from prospective data. Maternal and child health journal. 2013 Apr 1;17(3):493-500. | 1 |
| 1. Ameyaw EK. Prevalence and correlates of unintended pregnancy in Ghana: Analysis of 2014 Ghana Demographic and Health Survey. Maternal health, neonatology and perinatology. 2018 Dec;4(1):17. | 1 |
| 1. Geda NR, Lako TK. A population based study on unintended pregnancy among married women in a district in Southern Ethiopia. Journal of Geography and Regional Planning. 2011 Jul 31;4(7):417-27. | 2 |
| 1. Ameyaw EK. Prevalence and correlates of unintended pregnancy in Ghana: Analysis of 2014 Ghana Demographic and Health Survey. Maternal health, neonatology and perinatology. 2018 Dec;4(1):17. | 1 |
| 1. Ikamari L, Izugbara C, Ochako R. Prevalence and determinants of unintended pregnancy among women in Nairobi, Kenya. BMC pregnancy and childbirth. 2013 Dec;13(1):69. | 1 |
| 1. Hall JA, Barrett G, Phiri T, Copas A, Stephenson J. Prevalence and determinants of unintended pregnancy in Mchinji District, Malawi; using a conceptual hierarchy to inform analysis. PLoS One. 2016 Oct 31;11(10):e0165621. | 1 |
| 1. Mohamed EA, Hamed AF, Yousef FM, Ahmed EA. Prevalence, determinants, and outcomes of unintended pregnancy in Sohag district, Egypt. Journal of the Egyptian Public Health Association. 2019 Dec;94(1):14. | 1 |
| 1. Dutta M, Shekhar C, Prashad L. Level, trend and correlates of mistimed and unwanted pregnancies among currently pregnant ever married women in India. PloS one. 2015 Dec 2;10(12):e0144400. | 1 |
| 1. Yaya S, Amouzou A, Uthman OA, Ekholuenetale M, Bishwajit G, Udenigwe O, Hudani A, Shah V. Prevalence and determinants of terminated and unintended pregnancies among married women: analysis of pooled cross-sectional surveys in Nigeria. BMJ global health. 2018 Mar 1;3(2):e000707. | 1 |
| 1. Christofides NJ, Jewkes RK, Dunkle KL, McCarty F, Shai NJ, Nduna M, Sterk C. Risk factors for unplanned and unwanted teenage pregnancies occurring over two years of follow-up among a cohort of young South African women. Global health action. 2014 Dec 1;7(1):23719. | 1 |
| 1. Basinga P, Moore AM, Singh S, Remez L, Birungi F, Nyirazinyoye L. Unintended pregnancy and induced abortion in Rwanda: Causes and consequences. New York, NY: Guttmacher Institute. 2012. | 1 |
| 1. Logan C, Holcombe E, Manlove J, Ryan S. The consequences of unintended childbearing. Washington, DC: Child Trends and National Campaign to Prevent Teen Pregnancy. 2007 May;28:142-51. | 1 |
| 1. Yakubu I, Salisu WJ. Determinants of adolescent pregnancy in sub-Saharan Africa: a systematic review. Reproductive Health. 2018 Dec;15(1):15. | 1 |
| 1. Qian X, Tang S, Garner P. Unintended pregnancy and induced abortion among unmarried women in China: a systematic review. BMC health services research. 2004 Dec;4(1):1. | 1 |
| 1. Singh S, Sedgh G, Hussain R. Unintended pregnancy: worldwide levels, trends, and outcomes. Studies in family planning. 2010 Dec;41(4):241-50. | 1 |
| 1. Ali SA, Tikmani SS, Qidwai W. Prevalence and determinants of unintended pregnancy: systematic review. World Family Medicine Journal: Incorporating the Middle East Journal of Family Medicine. 2016 Jul;99(3671):1-0. | 1 |
| 1. Gerdts C, Dobkin L, Foster DG, Schwarz EB. Side effects, physical health consequences, and mortality associated with abortion and birth after an unwanted pregnancy. Women's Health Issues. 2016 Jan 1;26(1):55-9. | 1 |
| 1. Rahman MM, Rahman MM, Tareque MI, Ferdos J, Jesmin SS. Maternal pregnancy intention and professional antenatal care utilization in Bangladesh: a nationwide population-based survey. PloS one. 2016 Jun 16;11(6):e0157760. | 1 |
| 1. Bahk J, Yun SC, Kim YM, Khang YH. Impact of unintended pregnancy on maternal mental health: a causal analysis using follow up data of the Panel Study on Korean Children (PSKC). BMC pregnancy and childbirth. 2015 Dec;15(1):85. | 1 |

Reasons for exclusion: 1. Conducted in other countries; 2. Having data that were not extractable 3. The outcome of interests was not reported
