# Supplementary material for: Prevalence and determinants of unintended pregnancy in Ethiopia: A systematic review and meta-analysis of observational studies
Source: PLoS One. 2020 Apr 7;15(4):e0231012. doi: 10.1371/journal.pone.0231012 (PMC7138300; doi:10.1371/journal.pone.0231012)
Supplement: S2 Table — (DOCX) [file pone.0231012.s004.docx]

| **First author and publication year** | **Statistically significant variables with AOR(95%CI)** |
| --- | --- |
| Abame et al (2019) | **Effect of unintended pregnancy on ANC**  Intended: Ref.  Unintended: 0.31 (0.21,0.46) |
| Abayu et a l (2015) | **ANC visit Husband communication about pregnancy**  Yes: Ref. Yes: Ref.  No : 3.43(2.14,5.52) No: 2.25(1.43,3.52)  **Ever heard contraceptive** **Ever heard unintended pregnancy is preventable**  Yes: Ref. Yes: Ref.  No: 2.83(1.53,5.24) No: 2.56(1.58,4.16) |
| Admasu et al (2018) | **Current living condition Mass media available at home** **Marital status**  With husband: Ref. Yes: Ref. Single: Ref.  Living away from husband: 3.05 (1.46,6.34) No: 2.81 (1.27,6.23) Married: 0.18 (0.08,0.40) |
| Darega et al (2015) | **Age of the respondent TRENHF Decision making style**  20-29: 0.24 (0.06, 0.95) ≤ 30 Min: 0.68 (0.56, 0.80) Wife: Ref.  40-49: Ref. >60 min: Ref. Husband: 2.80 (1.38, 5.68)  **Educational level of the respondent Ever used any type of contraceptive method**  No formal education: Ref. Yes: 0.63 (0.39, 0.83)  Grade 10+: 0.31 (0.26, 0.66) No: Ref.  **Husband education Having child before Occupation of respondents**  No formal education: Ref. Yes: 3.91 (2.09, 7.31) Housewife: Ref.  10+: 0.53 (0.26, 0.89) No: Ref. Employee :0.79 (0.29, 0.75)  **Want any more children in future**  Yes: 0.58(0.36,0.95)  No: Ref. |
| Feyisso et al (2017) | **Women’s age Marital status of women Education level of women**  <20: 1.84(1.02, 4.29) Married: Ref. Illiterate: 0.07(0.02,0.26)  30-39: Ref. Single: 1.78(1.05,5.08) Junior /high school: 0.22(0.08,0.63)  Higher education: Ref. |
| Fite et al (2018) | **Age Marital status parity**  17-34: Ref. Married: Ref. 1: Ref.  35 and above: 2.343 (1.374, 3.997 single: 6.492 (1.299, 32.455) 2: 53.419 (21.453, 133.014  3 and above: 20.219 (7.915, 51.655)  **Abortion history** **Health professional visit** **Autonomy to choose contraceptive method**  Yes: Ref. Yes: Ref. Yes: Ref.  No: 1.962 (1.025, 3.755) No: 2.004 (1.218, 3.298 No: 2.925 (1.648, 5.190) |
| Gebreamlak et al (2014) | **Educational status of women Previous history of unintended pregnancy**  Illiterate: 3.10(1.66,5.78) Yes: 10.6(4.76,23.41)  Secondary and above: Ref. No: Ref.  **family size Partner awareness on contraceptive use**  1-2: Ref. Yes: Ref.  3-5: 2.19(1.32,3.61) No: 5.96(1.64,21.67)  >5: 8.90(4.37,18.13) |
| Gite et al (2016) | **Age at first marriage Knowledge on FP Husband refusal to use FP**  <18 years: 4.60(2.88, 7.34) Less knowledgeable: Ref. No: Ref.  ≥18 years: Ref. More knowledgeable: 0.40(0.075,0.37) Yes: 3.5 (1.97, 6.24) |
| Gizaw et al (2018) | **Residence number of children Discussion on FP**  Urban: 0.429(0.184, 0.996) 3-4: 0.502 (0.034, 0.921) yes: 0.033 (0.066, 0.177)  Rural: Ref. 5 and above: Ref. No: Ref.  **Distance from health facility** **knowledge about FP**  <30 minutes: 0.244(0.077, 0.768) Poor: 5.486 (1.178, 25.554)  30-60 minutes: 0.073(0.019, 0.287) Good: Ref.  ≥60 minutes: Ref. |
| Goshu et al (2019) | **Residence: Religion Educational status**  Urban: Ref. Orthodox: Ref. No formal education: Ref.  Rural: 2.6(1.5,4.6) Muslim: 0.79(0.6,0.90) Secondary school: 0.58(0.42,0.78)  **Gravidity parity**  1-2: Ref. 1-2: Ref.  3-4: 3.6(2.1,5.3) 3-4: 2.8 (2.6,9.7)  ≥5: 4.7(2.3,6.8) |
| Habte et al (2013) | **Age of women Ever use of FP Knows the timing of ovulation**  15-19: Ref. Yes:1.79 (1.31, 2.45) Yes: 0.55 (0.35, 0.85)  25-29: 0.46 (0.25, 0.83) No: Ref. No: Ref.  30-34: 0.40 (0.20, 0.78) **Numbers of children born No of deliveries in 5years**  **Education level** 0: Ref. 0: Ref.  No formal education: Ref. 5+: 2.36(1.01, 5.49) 2-4: 2.00 (1.12, 3.58)  Primary: 2.38 (1.73, 3.26) |
| Hamdela et al (2012) | **Previous unintended pregnancy Husband desire for family size Number of pregnancy**  Yes: 2.76(1.55, 4.91) Agree: Ref. 1-2: Ref. ≥5: Ref. Disagree: 3.24(1.69, 6.21) 3-4: 3.16(1.37, 7.3)  **Life time desired number of children** ≥5: 5.6(1.62, 19.41)  0-2: 5.86(1.83,18.76)  No: Ref. |
| Kahasay et al (2015) | **Stream School name Father’s life status**  Social: 0.46(0.23,0.92) Minilik: .11(0.03,0.39) Died: 2.79(1.57,4.94)  Natural: Ref. Ginibot 20: 0.16(0.04, 0.63) Alive: Ref.  Derartu: Ref.  **Mothers life status Mother’s educational status**  Died: 8.68(4.23, 17.80) Illiterate: Ref.  Alive: Ref. Secondary: 0.16(0.04,0.66)  College and more: 0.07(0.01,0.56) |
| Kassa et al (2012) | **Wealth status Time to walk to the nearest HF Parity**  Rich: Ref. <40 minutes: Ref. Never give birth: Ref.  Poor:1.47( 1.14, 1.9) 80 + minutes: 2.25( 1.49 ,3.39) 1-4: 2.32(1.59, 3.40)  5-6: 3.15( 2.05,4.86)  7 + birth: 5.182( 3.31,8.12) |
| Kassie et al (2017) | **Women’s education Spousal communication on FP Occupation**  Higher: Ref. Yes: Ref. Housewife: Ref.  Technical : 7.91(1.29,48.64) No: 3.92(1.61,9.54) Own business: 3.86(1.07,13.91)  **Awareness of long‐term FP**  Yes: Ref.  No: 4.46(1.04,19.08) |
| Kibret et al (2014) | **Marital status Ever use of FP** **Visited by health extension**  Married: Ref. Yes: Ref. Yes: Ref.  Never married:13.49(5.05,6.03) No: 1.51(1.50,6.45) No: 3.18(1.70,5.95)  **Occupation status Spousal communication MC Higher order of pregnancy**  Employee: Ref. Yes: Ref. <2: Ref.  Unemployment :2.26(1.71,7.61) No: 4.38(2.19,8.70) 5+ :7.45(2.27,24.44) |
| Melese et al (2016) | **Marital status Gravidity Distance to the nearest health facility**  Currently married: Ref. 1-2: Ref. >80 min.: 3.56 (1.69,7.53)  Formally married: 8.42 (4.07, 17.39) >5: 3.88 (1.41, 10.69) < 40 min.: Ref.  Never married: 9.21 (4.27, 19.86) **partner desire on children**  Agree: Ref.  Disagree: 4.09 (2.07, 8.08) |
| Mohammed et al (2016) | **Marital status Parity Heard about contraceptives**  Married: Ref. ≤2: Ref. Yes: Ref.  Single: 5.5(2.25, 13.64) 3-5: 2.37(1.36, 4.15) No: 2.73(1.15, 6.50)  Divorced/widowed: 4.0 (1.31, 12.45) 5+: 4.76(2.40, 9.65) |
| Mulat et al (2017) | **Women age Education level Occupation**  15-19: 3.68(1.03,13.16) illiterate: 4.55(1.78,11.63) unemployed: 0.03(0.004,0.166)  30-34: 0.12(0.02, 0.80) preparatory: 0.15(0.02, 0.88) others: Ref.  35-39: 0.07(0.013, 0.41) Diploma and above: Ref.  ≥40: Ref.  **Marital status** **Ever use of FP**  Single: 92.95(2.8, 31.05) Yes: Ref.  Widowed: Ref. No: 0.14(0.06,0.3) |
| Tebekaw et al (2014) | **Age of women Parity Marital status**  15-19: 1.66 (1.27, 2.17) 0-2: Ref. Never in union: Ref.  20-34: Ref. 3-5: 1.29 (1.09,1.52) Married/cohabiting: 0.12 (0.07, 0.22)  **Woman's educational** 6+: 1.56 (1.25,1.94) formally married :0.20 (0.11, 0.36)  No education: Ref. **Household size Ethnicity**  Secondary education: 0.63 (0.47, 0.84) 1-3: Ref. Oromo: Ref.  **Currently working** 4-6: 1.27 (1.04, 1.55 **)** Afar:0.76 (0.63, 0.91)  Yes: 1.23 (1.09, 1.40) 7+: 1.74 (1.39, 2.18) Amhara: 0.15 (0.10, 0.23)  No: Ref. **Religion** Somali: 0.13 (0.09, 0.19)  **Wealth index** Orthodox: Ref. Tigrie: 0.37 (0.29, 0.47)  Rich: 1.29 (1.10, 1.51) Protestant: 0.76 (0.62,0.93) Others: 0.62 (0.53, 0.73)  middle: Reference category Muslim: 0.77 (0.65,0.91)  **Knowledge of contraceptive method Contraceptive use Decision making on HC**  Knows no method: Ref. Yes: Ref. Alone: Ref.  Knows at least one method: 1.82 (1.25,2.65) No: 0.64(0.56, 0.73) Joint : 0.74 (0.62,0.88) |
| Teshome et al (2010) | **Age of respondents Husband desire Number of birth**  20-24: 0.42(0.24, 0.73) Agree: Ref. None: Ref.  25-29: Ref. Disagree: 2.26(1.23, 4.14) 3-4: 2.01(1.01, 3.99)  30-34:2.21(1.13, 4.15) **Ever physical violated** ≥5: 3.03(1.13, 8.06)  35-45:6.51(2.73, 15.5) Yes: 1.78(1.18, 2.70) **FP worker visited**  **Desired number of children No:** Ref. Yes: 0.63(0.42, 0.95)  0-2: 7.03(3.37, 14.7) **Age at marriage** No: Ref.  3-4: 5.14(2.89, 9.12) <18: 4.60(2.88, 7.34) **Knowledge related to FP**  ≥5: Ref. ≥18: Ref. Low knowledge: 3.76(2.37, 5.96)  High knowledge: Ref. |
| Tsegaye et al (2018) | **History of still birth Age Discussing pregnancy related issue with husband**  Yes: 3.3 (1.4, 7.9) 15-19: Ref. Yes: Ref.  No: Ref. 40-44: 15.2 (1.9, 125.2) No: 2.3 (1.1, 5.0)  **Decision on FP**  Husband: Reference  Myself: 0.4 (0.2, 0.8)  Both: 0.2 (0.1, 0.4) |
| Wado et al (2013) | **Use of ANC (outcome variable) Receiving adequate vs inadequate ANC (outcome)**  **pregnancy intention** **pregnancy intention**  Intended: Ref. Intended: Ref.  unintended: 0.75 (0.58,0.97) unintended: 0.67 (0.46,0.96) |
| Weldegebreal et al (2015) | **Ever gave birth before unintended pregnancy happened** **Had steady partner**  Yes: 3.07 (1.54, 6.09) Yes: 2.87 (1.47, 5.61)  No: Ref. No: Ref.  **Ever abort before unintended pregnancy happened** **Drug user** **duration of sex work**  Yes: 15.64 (8.03, 30.47 yes: 2.68 (1.30, 5.52) <12 months: Ref.  No: Ref. No: Ref. 60–96 months: 0.33 (0.11, 0.95) |
| Worku et al (2006) | **Age Education Age at first marriage**  15-19: 4.23(1.42,10, 54) primary: 1.65(1.01, 2.6) 10-14: 2.05(1.89,4.74)  25-29: 0.54(0.29, 0.98) secondary and higher: Ref. 15-19: 1.38(1.75,2.56)  40-49:Ref. 25 and above: Ref.  **Marital status Number of pregnancies**  Married: Ref. ≤2: 0.55(0.33,0.74)  Single: 1.72(1.20,2.47) 3-4: 0.57(0.37,0.89)  5 and above: Ref. |
| Yenealem et al (2019) | **Family size** **Age at first pregnancy** **Marital status**  ≥4: 2.92 (1.605, 5.314 <18: 3.025 (1.234, 6.056) Single: 12.592 (5.182, 30.6)  <4:Ref. ≥18:Ref. Married: Ref. |
| Liyew et al | **Husband education Previous unintended pregnancy Spousal communication on FP**  Technical / vocational: 1.60(1.36,10.32) Yes: 3.84(1.15,12.83) Yes:0.32(0.10,0.98)  Unable to read and write: Ref. No: Ref. No: Ref.  **Using of FP according to instruction Household money income**  Yes:0.09(0.02,0.36) 501-1000: 0.13(0.02,0.86)  No: Ref. >3000: Ref. |

*ANC=antenatal care, AOR=adjusted odds ratio, CI=confidence interval, MC=modern contraceptive, HF=health facility, Ref. =reference, TRENHF= Time respondents elapse to the near health facility providing contraceptives*
