# Supplementary material for: Prevalence and determinants of unintended pregnancy in Ethiopia: A systematic review and meta-analysis of observational studies
Source: PLoS One. 2020 Apr 7;15(4):e0231012. doi: 10.1371/journal.pone.0231012 (PMC7138300; doi:10.1371/journal.pone.0231012)
Supplement: Attachment — Submitted filename: PONE-D-19-21662_reviewer (1).pdf [file pone.0231012.s005.pdf]

PLOS ONE

Prevalence and determinants of unintended pregnancy in Ethiopia: a systematic review and meta-analysis of observational studies

--Manuscript Draft--

Manuscript Number: PONE-D-19-21662

Article Type: Research Article

Full Title: Prevalence and determinants of unintended pregnancy in Ethiopia: a systematic review and meta-analysis of observational studies

Short Title: Unintended pregnancy in Ethiopia

Corresponding Author: Muluneh Alene Addis, MPH
Debre Markos University
Debre Markos, ETHIOPIA

Keywords: Magnitude, Unplanned pregnancy, Ethiopia, Meta-analysis, Mothers

Abstract:

Abstract

Background: Pregnancies should be planned before conception unless a woman may not be in optimal health for childbearing. Despite, a number of studies with inconsistent findings were conducted to reduce unintended pregnancy in Ethiopia; unavailability of nationwide study that determines the prevalence of unintended pregnancy and its determinants is an important gap. Thus, this study was conducted to determine the overall prevalence of unintended pregnancy and its determinants in Ethiopia.

Methods: We searched from Google scholar, PubMed, and Science Direct data bases for studies. The risk of bias for each of the original study was assessed using the existing tool of risk of bias assessment for observational studies. The heterogeneity of studies was also assessed using I2 test statistics. Data were pooled and a random effect meta-analysis model was fitted to provide the overall prevalence of unintended pregnancy and its determinants in Ethiopia. In addition, the subgroup analyses were performed to investigate how the prevalence of unintended pregnancy varies across different groups of studies.

Results: Twenty seven studies which satisfy the eligibility criteria were included. We found that the overall prevalence of unintended pregnancy in Ethiopia was 28% (95% CI: 25-31). The subgroup analysis showed that the highest prevalence of unintended pregnancy was observed from Oromiya region (33.8%) followed by SNNPR (30.6%), and the lowest was in Harar. In addition, the pooled prevalence of unintended pregnancy was 26.4% (20.8-32.4) and 30.0% (26.6-33.6) for community based cross sectional and institution based cross sectional studies respectively. The pooled analysis showed that not communicating with husband about family planning were more likely to have unintended pregnancy (OR: 3.56, 95%CI: 1.68-7.53). The pooled odds ratio also showed that unintended pregnancy is more likely among women who never use family planning method (OR: 2.08, 95%CI: 1.18-3.69). Furthermore, the narrative review of this study showed that maternal education, age and household wealth index were strongly associated with unintended pregnancy.

Conclusions: In this study, the prevalence of unintended pregnancy was high. Lack of spousal communication, never use family planning, maternal education and household wealth level were significantly associated with unintended pregnancy. This study implies ~~to~~ the need for plans and policies to improve the awareness of contraceptive utilization and strengthen spousal communication related to pregnancy.

Order of Authors:

Muluneh Alene Addis, MPH

Leltework Yismaw Wondemagegn

Yebelay Berelie Berhan

Bekalu Kassie Alemu

Reta Yeshamble Kessete

Moges Agazhe Assemie

Additional Information:

Powered by Editorial Manager® and ProduXion Manager® from Aries Systems Corporation

Question	Response
**Financial Disclosure**<br><br>Enter a financial disclosure statement that describes the sources of funding for the work included in this submission. Review the submission guidelines for detailed requirements. View published research articles from *PLOS ONE* for specific examples.<br><br>This statement is required for submission and **will appear in the published article** if the submission is accepted. Please make sure it is accurate.<br><br>**Unfunded studies**<br>Enter: *The author(s) received no specific funding for this work.*<br><br>**Funded studies**<br>Enter a statement with the following details:<br>• Initials of the authors who received each award<br>• Grant numbers awarded to each author<br>• The full name of each funder<br>• URL of each funder website<br>• Did the sponsors or funders play any role in the study design, data collection and analysis, decision to publish, or preparation of the manuscript?<br>• **NO** - Include this sentence at the end of your statement: *The funders had no role in study design, data collection and analysis, decision to publish, or preparation of the manuscript.*<br>• **YES** - Specify the role(s) played.<br><br>* typeset	The author(s) received no specific funding for this work.
**Competing Interests**<br><br>Use the instructions below to enter a competing interest statement for this submission. On behalf of all authors, disclose any competing interests that could be perceived to bias this work—acknowledging all financial support and any other relevant financial or non-financial competing interests.<br><br>This statement **will appear in the published article** if the submission is	The authors have declared that no competing interests exist.

accepted. Please make sure it is accurate. View published research articles from *PLOS ONE* for specific examples.<br><br>**NO authors have competing interests**<br><br>Enter: *The authors have declared that no competing interests exist.*<br><br>**Authors with competing interests**<br><br>Enter competing interest details beginning with this statement:<br><br>*I have read the journal's policy and the authors of this manuscript have the following competing interests: [insert competing interests here]*<br><br>* typeset	
**Ethics Statement**<br><br>Enter an ethics statement for this submission. This statement is required if the study involved:<br><br>• Human participants<br>• Human specimens or tissue<br>• Vertebrate animals or cephalopods<br>• Vertebrate embryos or tissues<br>• Field research<br><br>Write "N/A" if the submission does not require an ethics statement.<br><br>General guidance is provided below. Consult the submission guidelines for detailed instructions. **Make sure that all information entered here is included in the Methods section of the manuscript.**	N/A

**Format for specific study types**<br><br>**Human Subject Research (involving human participants and/or tissue)**<br>• Give the name of the institutional review board or ethics committee that approved the study<br>• Include the approval number and/or a statement indicating approval of this research<br>• Indicate the form of consent obtained (written/oral) or the reason that consent was not obtained (e.g. the data were analyzed anonymously)<br><br>**Animal Research (involving vertebrate animals, embryos or tissues)**<br>• Provide the name of the Institutional Animal Care and Use Committee (IACUC) or other relevant ethics board that reviewed the study protocol, and indicate whether they approved this research or granted a formal waiver of ethical approval<br>• Include an approval number if one was obtained<br>• If the study involved *non-human primates*, add *additional details* about animal welfare and steps taken to ameliorate suffering<br>• If anesthesia, euthanasia, or any kind of animal sacrifice is part of the study, include briefly which substances and/or methods were applied<br><br>**Field Research**<br><br>Include the following details if this study involves the collection of plant, animal, or other materials from a natural setting:<br>• Field permit number<br>• Name of the institution or relevant body that granted permission	
**Data Availability**<br><br>Authors are required to make all data underlying the findings described fully available, without restriction, and from the time of publication. PLOS allows rare exceptions to address legal and ethical concerns. See the PLOS Data Policy and FAQ for detailed information.	Yes - all data are fully available without restriction

Powered by Editorial Manager® and ProduXion Manager® from Aries Systems Corporation

A Data Availability Statement describing where the data can be found is required at submission. Your answers to this question constitute the Data Availability Statement and **will be published in the article**, if accepted.<br><br>**Important:** Stating 'data available on request from the author' is not sufficient. If your data are only available upon request, select 'No' for the first question and explain your exceptional situation in the text box.<br><br>Do the authors confirm that all data underlying the findings described in their manuscript are fully available without restriction?	
**Describe where the data may be found in full sentences. If you are copying our sample text, replace any instances of XXX with the appropriate details.**<br><br>• If the data are **held or will be held in a public repository**, include URLs, accession numbers or DOIs. If this information will only be available after acceptance, indicate this by ticking the box below. For example: *All XXX files are available from the XXX database (accession number(s) XXX, XXX.*).<br>• If the data are all contained **within the manuscript and/or Supporting Information files**, enter the following: *All relevant data are within the manuscript and its Supporting Information files.*<br>• If neither of these applies but you are able to provide **details of access elsewhere**, with or without limitations, please do so. For example:<br><br>*Data cannot be shared publicly because of [XXX]. Data are available from the XXX Institutional Data Access / Ethics Committee (contact via XXX) for researchers who meet the criteria for access to confidential data.*<br><br>*The data underlying the results presented in the study are available from (include the name of the third party*	All relevant data are within the manuscript and its Supporting Information files.

and contact information or URL).<br>• This text is appropriate if the data are owned by a third party and authors do not have permission to share the data.<br><br>* typeset	
Additional data availability information:	

Manuscript Click here to download Manuscript Manuscript.docx

Prevalence and determinants of unintended pregnancy in Ethiopia: a systematic review and meta-analysis of observational studies

Muluneh Alene[1*], Leltework Yismaw[1], Yebelay Berelie[2], Bekalu Kassie[3], Reta Yeshambel[4], Moges Agazhe Assemie[1]

1Department of Public Health, Debre Markos University, Debre Markos, Ethiopia
2Department of Statistics, Debre Markos University, Debre Markos, Ethiopia
3Department of Midwifery, Debre Markos University, Debre Markos, Ethiopia
4Department of Biology, Mizan-Tepi University, Teppi, Ethiopia

Author's email addresses
MA: mulunehadis@gmail.com
LY: lielt.yismaw@gmail.com
YB: yebelay.ma@gmail.com
BK: bekalukassiedmu@gmail.com
RY: reta.yeshambel@gmail.com
MAA: agazhemoges@gmail.com

***Corresponding author**
mulunehadis@gmail.com

Abstract

Background: Pregnancies should be planned before conception unless a woman may not be in optimal health for childbearing. Despite, a number of studies with inconsistent findings were conducted to reduce unintended pregnancy in Ethiopia; unavailability of nationwide study that determines the prevalence of unintended pregnancy and its determinants is an important gap. Thus, this study was conducted to determine the overall prevalence of unintended pregnancy and its determinants in Ethiopia**.**

Methods: We searched from Google scholar, PubMed, and Science Direct data bases for studies. The risk of bias for each of the original study was assessed using the existing tool of risk of bias assessment for observational studies. The heterogeneity of studies was also assessed using I^2 test statistics. Data were pooled and a random effect meta-analysis model was fitted to provide the overall prevalence of unintended pregnancy and its determinants in Ethiopia. In addition, the subgroup analyses were performed to investigate how the prevalence of unintended pregnancy varies across different groups of studies.

Results: Twenty seven studies which satisfy the eligibility criteria were included. We found that the overall prevalence of unintended pregnancy in Ethiopia was 28% (95% CI: 25-31). The subgroup analysis showed that the highest prevalence of unintended pregnancy was observed from Oromiya region (33.8%) followed by SNNPR (30.6%), and the lowest was in Harar. In addition, the pooled prevalence of unintended pregnancy was 26.4% (20.8-32.4) and 30.0% (26.6-33.6) for community based cross sectional and institution based cross sectional studies respectively. The pooled analysis showed that not communicating with husband about family planning were more likely to have unintended pregnancy (OR: 3.56, 95%CI: 1.68-7.53). The pooled odds ratio also showed that unintended pregnancy is more likely among women who never use family planning method (OR: 2.08, 95%CI: 1.18-3.69). Furthermore, the narrative review of this study showed that maternal education, age and household wealth index were strongly associated with unintended pregnancy.

Conclusions: In this study, the prevalence of unintended pregnancy was high. Lack of spousal communication, never use family planning, maternal education and household wealth level were significantly associated with unintended pregnancy. This study implies to the need for plans and

policies to improve the awareness of contraceptive utilization and strengthen spousal communication related to pregnancy.

Key words: Magnitude, Unplanned pregnancy, Ethiopia, Meta-analysis, Mothers

Introduction

Unintended pregnancy is a pregnancy which is either mistimed or unwanted (1,2). It is a public health problem and a risk factor for adverse health outcomes, particularly for maternal and child health (3). Though the rate of unintended pregnancy fell worldwide, it dropped less sharply in developing regions than in developed regions (3). Ethiopia is one of the developing countries with high prevalence of unintended pregnancy. In Ethiopia, more-than one-third (38%) of pregnancies were unintended in 2014 that is slightly lower than in 2008 which was 42%. According to the 2016 Ethiopian Demographic and Health Survey (EDHS) result, of all births in the past five years and current pregnancies, 25% was unintended. In addition, the 2016 EDHS report showed that the overall difference between the wanted fertility rate and the total fertility rate is one child, which suggests that Ethiopian women are currently having, on average one child more than they want (4).

Pregnancies should be planned before conception unless a woman may not be in optimal health for childbearing (2). Unintended pregnancy leads to maternal mortality and morbidity due to the complications of unsafe abortion, miscarriage and unplanned births, which burdens the health system at all (5–7). Annually, more than 1 in 10 pregnancies and 1 in 27 mothers end in abortion and die due to the complications of pregnancy or childbirth in Ethiopia respectively (8). A woman with unintended pregnancy will have low physical and mental health, low self-care, and depression during pregnancy. These leads for poor Antenatal Care (ANC) service utilization and postpartum depression, which is a risk factor for unfavorable pregnancy outcome, maternal morbidity and mortality (7,9). Consequently, the newborns of unintended pregnancies are faced for low birth weight and inadequate vaccinations which are a risky for childhood illnesses (5,7,10–12). Previous evidences reported that unintended pregnancy mainly results from not properly using modern contraception (inconsistent or incorrect use of contraceptive methods) and women are less likely than men to want more children no matter how many children they already have (2,13).

In Ethiopia, a number of studies were conducted to estimate the magnitude and ~~to~~ identify the determinant ~~factors~~ of unintended pregnancy. However, the reported prevalence and determinants in these fragmented studies vary depending on the characteristics of study participants, the type of design employed and variables analyzed. Having conclusive evidence by combing the existing studies is significant to have quality evidence on the national prevalence and determinants of unintended pregnancy. Thus, this systematic review and meta-analysis was conducted to determine the overall prevalence and determinants of unintended pregnancy in Ethiopia. This review will bring an improvement on the design of future studies related to unintended pregnancy. The findings of this study will also ~~highlight the prevalence and determinant factors of unintended pregnancy to~~ improve health workers interventions ~~working~~ in the area of reproductive health.

Materials and methods

Study design and setting

A systematic review and meta-analysis, which aimed to estimate the overall prevalence of unintended pregnancy and its determinants was conducted in Ethiopia. Ethiopia is situated in the horn of Africa, ~~and~~ bordered by Eritrea to the north, Sudan and South Sudan to the west, Kenya to the south, and Djibouti and Somalia to the east. In Ethiopia, nearly eight in ten women (78%) and half of women age 15-49 (48%) are living in rural areas and have no education respectively (4).

Eligibility criteria

In this systematic review studies were included with the following criteria: 1) only studies conducted in Ethiopia. 2) Only studies reported in English language. 3) Only studies involving pregnant women or had given birth at least once preceding the survey. 4) All observational studies reporting the prevalence and determinants of unintended pregnancy. ~~5)~~ Both published and unpublished articles were included. Studies which were not fully accessible were excluded because of the inability to assess the quality of studies without of full text.

Searching for studies

Comprehensive search strategy was done by three (MA, LY and RY) of the authors. Both published and unpublished articles on unintended pregnancy were searched from ~~international~~

~~and national electronic databases. We searched from~~ international (Google scholar, PubMed, and Science Direct), and national electronic data bases ~~for studies.~~ First, articles were searched by examining the full titles ("Prevalence and determinants of unintended pregnancy in Ethiopia") and then keywords (unintended pregnancy, unplanned pregnancy, unwanted pregnancy, mistimed pregnancy, determinants, risk factors, associated factors, Ethiopia). These keywords were used separately and in combination using Boolean operators "OR" or "AND". In addition, we ~~were~~ searched from the reference lists of all the included studies (snowball technique) to identify any other studies that may have been missed by our search strategy. Finally, all studies were imported into reference management software (Mendeley Desktop).

Outcome measures and data extraction

Unintended pregnancy which is either mistimed or unwanted was the primary outcome of the study. Unwanted pregnancy occurred when a woman did not want to have any more pregnancies, whereas mistimed pregnancy is a pregnancy that was wanted by the woman at some time, but which occurred sooner than they want. The pregnancy intention for the included studies was assessed by interviewing current pregnant women or had given birth at least once preceding the survey.

All essential data from the included studies were extracted independently by two (MA and LY) of authors using a predesigned data abstraction form. This form includes the last name of the first author, publication year, data collection period, study design, region of the study conducted, study population, sample size, response rate, and the magnitude of unintended pregnancy. In addition, we extracted the adjusted odds ratios with corresponding 95% confidence intervals to measure the strength of effects. ~~The~~ deviation in a data extraction process was resolved by discussion and consensus involving all authors.

Quality assessment for studies

The quality of meta-analysis depends on the included studies (14). Two authors (MA and YB) assessed ~~the~~ risk of bias for the included studies using the ~~existing~~ tool of risk of bias assessment for observational studies (15). This tool includes 10 items. The first four items assess the external validity, while the other six items evaluates the internal validity of the study. All items of the tool was filled for each included studies and categorized as low risk (if the response is "yes"), higher risk (if the response is "no"), and not clear. The quality of the study was examined by summing

the score given for each items. Lastly, not clear risk of bias was categorized as high risk of bias ~~then;~~ the summary assessment risk of bias for each study was categorized according to the number of high risk of bias ~~per study~~ low (≥2), moderate (3-4), and high (≥5).

Data processing and analysis

After extracting all relevant data using Microsoft excel software, it was exported to R statistical software for Meta-analysis. The double arcsine transformation which stabilizes the sampling variance was applied to estimate the weighted average prevalence, and the transformed summary prevalence ~~are~~ converted back for ease of interpretation (16). We assessed the consistency of studies using I^2 test statistics (17). This test examines the null hypothesis that all the included studies are evaluating the same effect. Consequently, since there was heterogeneity between the original studies (I^2=96%, p<0.01) a random effect model is needed. Therefore, to account this between study variance a random effect meta-analysis with an estimation of DerSimonian and Laird method was performed. Furthermore, subgroup analyses were conducted to investigate how the prevalence of unintended pregnancy varies across different subgroups of studies.

Results

Search results

Our comprehensive search for studies was between 15th February and 30th April 2019. The flow chart diagram that shows our literature search, study selection and the number of included studies is shown in (**Fig 1**). First, a total of 273 articles were identified during our search, and then 115 articles were excluded due to duplication. Finally, 27 studies which satisfied the eligibility criteria were included in this systematic review and meta-analysis.

Figure 1: Flow chart diagram describing the selection of studies included in the systematic review and meta-analysis of prevalence and determinants of unintended pregnancy in Ethiopia.

Description of the included studies

The detail descriptions of the included studies are shown in (**Table 1**). All twenty seven studies were conducted between 2006 and 2019. Of all the included studies, eleven (10,18–27) were community based cross sectional, while twelve were institutional based cross sectional studies (28–38). The data for two studies were also taken from the 2011 EDHS (39,40). The majority of respondents of the original studies were pregnant mothers. The number of study participants

~~were~~ varied from the lowest 165 (28) to the highest 7,759 (40). Accordingly, a study with the smallest study participants was institutional based cross-sectional study, while the data for the highest study participants were taken from the 2011 EDHS. Furthermore, nine studies were conducted in Amhara region, six studies in Oromiya region, six studies in SNNPR, two studies in Addis Ababa, one study in Harar, and two studies from the national survey. Moreover, the prevalence of unintended pregnancy ranged from 13.7% (26) to 41.5% (22).

Table 1: Descriptions of the included studies conducted in Ethiopia on unintended pregnancy.

**First author (publication year)**	**Study area (Region)**	**Study design**	**Data collection period**	**Study population**	**Sample size**	**Response rate**	**Prevalence of unintended pregnancy**
Abame et al (2019)	SNNPR	CBCS	March 13, 2017 to April 13, 2017	pregnant mothers	748	97%	36.2%
Abayu et al (2015)	Tigray	CBCS	24/09/2012 to 18/10/2012	Pregnant women	626	96.3%	26%
Admasu et al (2018)	Amhara	CBCS	NR	pregnant women or with under 1 child age	680	91%	15.8%
Ayele et al (2017)	Oromiya	IBCS	June 10, 2017 to July 24, 2017	pregnant women following ANC	165	96%	35.2%
Darega et al (2015)	Oromiya	IBCS	May ,2014	Women following ANC	362	100%	37.3%
Feyisso et al (2017)	SNNPR	IBCS	February to June, 2016	Women following ante natal and post	290	NR	36.9%

				natal ANC			
Fite et al (2018)	Oromiya	CBCS	May 01–July 30, 2017	Pregnant women	704	91.5%	41.5%
Gebreamlak et al (2014)	Amhara	IBCS	June to July 2012	Pregnant women	454	NR	26%
Gite et al (2016)	SNNPR	CBCS	February 15-March 11,2015	Pregnant women	311	95.4%	19.4%
Gizaw et al (2018)	SNNPR	IBCS	February 24 to April 24, 2017	Pregnant women	224	100%	22.3%
Goshu et al (2019)	Amhara	IBCS	April 01 to May 30, 2018	Pregnant women	398	100%	26.1%
Habte et al (2013)	National level	EDHS	NA	Pregnant women	1267	NA	24%
Hamdela et al (2012)	SNNPR	CBCS	April 02 to 15, 2011	pregnant married women	385	100%	34%
Kahasay et al (2015)	Addis Abeba	IBCS	NR	female students aged from 16-19 years	576	100%	20.4%
Kassa et al (2012)	Oromiya	KDSHRC	December 2009 to November	Pregnant women	2072	100%	27.9%

			2010				
Kassie et al (2017)	Addis Abeba	IBCS	February to May 2015	Pregnant women	393	100%	36.4%
Kibret et al (2014)	Amhara	IBCS	April 15 to May 14, 2012	Pregnant women	413	100%	32.9%
Liyew et al (2017)	Amhara	IBCS	NR	Pregnant women	285	100%	28.4%
Melese et al (2016)	Amhara	CBCS	NR	Pregnant women	690	NR	23.5%
Mohammed et al (2016)	Oromiya	IBCS	January 10 to April 13, 2015	Pregnant women	413	97.9%	27.1%
Mulat et al (2017)	SNNPR	IBCS	NR	Pregnant women	362	100%	33.7%
Tebekaw et al (2014)	National level	EDHS	EDHS, 2011	Women who had at least one birth	7,759	NA	32%
Teshome et al (2010)	Amhara	CBCS	NR	currently married women	576	NR	40.8%
Tsegaye et al (2018)	Amhara	CBCS	August to September 2015	Married pregnant women	619	95.6%	13.7%
Wado et al	Oromiya	HDSS	March,2012	mothers	1456	94%	35%

(2013)				with alive birth in the two years			
Worku et al (2006)	Harar	CBCS	November to December 2001	Reproductive age women	983	98.3%	33.3%
Yenealem et al (2019)	Amhara	CBCS	April 1–May 30, 2014	Pregnant women	325	100%	20.6%

CBCS= Community Based Cross Sectional Study, EDHS= Ethiopia Demographic and Health Survey, HDSS=Health and Demographic Surveillance System, IBCS=Institution Based Cross Sectional Study, KDS-HRC= Kersa Demographic Surveillance and Health Research Center, NA= Not Applicable, NR=Not Report, SNNRP= Southern Nations, Nationalities, and Peoples' Region

Risk of bias for the included studies

We assessed the risk of bias for each of the original study using the existing risk of bias assessment tool (**Table S1**). Accordingly, of the total included studies, our summary assessment showed that more than half (55.6%) of the studies had low risk of bias, and nearly one-fourth (25.9%) of studies had moderate risk of bias. Finally, less than one-fifth (18.5%) of the included studies had a high risk of bias.

Prevalence of unintended pregnancy in Ethiopia

In this study, the overall prevalence of unintended pregnancy in Ethiopia was 28% (95% CI: 25-31) (**Fig 2**). We had conducted subgroup analyses to investigate how the prevalence of unintended pregnancy varies across different subgroups of studies (**Table 2**). Consequently, the pooled prevalence of unintended pregnancy was 26.4% (20.8-32.4) and 30.0% (26.6-33.6) for community based cross sectional and institution based cross sectional studies respectively. The subgroup analysis based on the region where studies were conducted also showed that the highest prevalence of unintended pregnancy was observed from Oromiya region [33.8% (29.0-38.7)] followed by SNNPR [30.6% (25.2-36.2)] and Addis Ababa [28.0% (14.0-44.7)]. The lowest prevalence was also noted in Harar [22.9% (20.3-25.5)]. Furthermore, the pooled

prevalence of unintended pregnancy was 28.2% (25.1-31.4) and 28.8% (23.7-34.0) for studies conducted prior and post 2014 respectively.

Fig 2. Forest plot of the pooled prevalence of unintended pregnancy in Ethiopia

Table 2: Subgroup analysis of studies included in meta-analysis on prevalence and determinants of unintended pregnancy in Ethiopia

**Subgroup**	**Random effects (95%CI)**	**Test of heterogeneity (I^2)**
**By study design**		
CBCS	26.4% (20.8-32.4)	96.6%
IBCS	30.0% (26.6-33.6)	84.4%
Overall	28.2% (24.1-32.5)	82.5%
**By region**		
Amhara	24.9% (19.2-31.0)	95.2%
Oromiya	33.8% (29.0-38.7)	91.2%
SNNPR	30.6% (25.2-36.2)	87.6%
Addis Ababa	28.0% (14.0-44.7)	96.6%
National level	28.0% (20.5-36.1)	97.1%
Tigray	26.5% (23.1-30.0)	-
Harar	22.9% (20.3-25.5)	-
Overall	26.5%(24.8-28.2)	88.8%
**By data collection period**		
2014 and before	28.2% (25.1-31.4)	94.6%
After 2014	28.8% (23.7-34.0)	94.5%
Overall	28.4% (25.7-31.1)	87.2%

We assessed the issue of publication bias by visual inspection of funnel plot and using the Egger's regression test. Though the funnel plot looks asymmetrical (**Fig 3**), the Egger's test showed ~~that~~ no relationship between the effect size and its precision (P = 0.776). This might be due to heterogeneity in true effects.

Fig 3: Funnel plot of the prevalence of unintended pregnancy in Ethiopia.

Determinants of unintended pregnancy in Ethiopia

The significant determinants of unintended pregnancy reported from each study are presented in (**Table S2**). Of twenty-one studies which examined the association of maternal education level with unintended pregnancy, ten studies reported that maternal education status was associated with unintended pregnancy (29,31,32,34–36,38–40). However, we couldn't pool quantitatively because of inconsistent classification of maternal education level.

In six studies researchers examined the association between spousal communication about family planning and unintended pregnancy. Of this, five studies (26,30,36,37,41) showed that spousal communication about family planning were associated with unintended pregnancy. The result of this meta-analysis showed that not communicating with husband about family planning were more likely to have unintended pregnancy (OR: 3.56, 95%CI: 1.68-7.53) (**Fig 4**). Ten studies ~~were~~ examined the association between the use modern family planning method and unintended pregnancy(10,22,26,29,34,37–39,41,42). Consequently, the result of this study showed that unintended pregnancy is more likely among women who never used family planning method (OR: 2.08, 95%CI: 1.18-3.69) (**Fig.5**).

Fig 4. The pooled odds ratio of the association between spousal communication about family planning and unintended pregnancy in Ethiopia.

Fig 5. The pooled odds ratio of the association between the use of family planning and unintended pregnancy in Ethiopia.

Discussion

Unintended pregnancy has ~~a~~ significant consequences for the health and welfare of women and children (7). ~~The~~ pregnancy intention in developing countries are influenced by socio-cultural, environmental, individual and health service related factors (43). This systematic review and meta-analysis was conducted to determine the overall prevalence of unintended pregnancy and to identify its determinants in Ethiopia**.**

Accordingly, we found that the overall prevalence of unintended pregnancy in Ethiopia was 28% (95%CI: 25-31). This finding is consistent with the 2016 EDHS result (4), which reported that from all births in the past 5 years and current pregnancies, one-fourth (25%) ~~of them~~ were unintended. Additionally, in the previous two consecutive EDHS reports, we noted that the proportion of women who want no more births ~~was~~ declined from 42% in 2005 to 37% in 2011.

The prevalence of unintended pregnancy found in this study was in agreement with other previous studies conducted in Egypt (44) , Ghana (45), and Bangladesh (9) , which reported that nearly one-third (30.7%), 29.8% and 29% of pregnancies were unintended respectively. However, the magnitude of unintended pregnancy found in this study was lower than previous studies conducted in Malawi, South Africa, and republic of Congo (46–49). This variation might be attributed to methodological difference in the assessment of pregnancy intention. The other possible reason might be inconsistency in the prevalence of unintended pregnancy could be explained by the difference in the socio-economic characteristics of study participants (50–52).

In this study, we found that the pooled prevalence of unintended pregnancy was 26.4% and 30% for community based cross sectional and institutional based cross sectional studies respectively. This variation could be explained by institution based studies will include women who visit health facilities for legal abortion services, which is probably unintended pregnancy. About 97 % of women seeking an abortion reported having an unintended pregnancy (50). The highest prevalence of unintended pregnancy was observed from Oromiya region (33.8%), and the lowest was in Harar (22.9%). The possible explanation might be due to the difference in the utilization of family planning method.

In this systematic review and meta-analysis, we noted that a number of factors were found to be associated with unintended pregnancy. Consequently, we found that women's who hadn't communication with their husband about family planning method were more likely to have unintended pregnancy. The possible explanation for this result might be women's perception that their husbands oppose family planning is one of the dominant factors for discouraging contraceptive practice in a wide variety of settings (20). Partner awareness on contraceptive and open discussion about family planning method will decreased the risk of unintended pregnancy. The result of this study also showed that unintended pregnancy is more likely among women who never used family planning method. This is due to the fact that awareness and proper utilization of modern contraceptive is crucial for reducing unintended pregnancy.

In this systematic review, unintended pregnancy was less likely among married women. The possible reason for this result might be in Ethiopian culture pregnancy without married is unacceptable in most of the communities. This result is supported by previous studies conducted in South Africa and Kenya (53,54).

Though previous evidences stated that youngest women experience the highest rate of unintended pregnancy (55), in this systematic review, we observed controversial results on the association between women's age and unintended pregnancy. Some of the studies reported that younger women's are more likely to have unintended pregnancy, while other studies showed that older women's are more likely to have unintended pregnancy.

Due to inconsistent classification of maternal education level, we couldn't pool quantitatively the association between maternal education and unintended pregnancy. But, contradictory results are observed between individual studies. Accordingly, four studies (29,32,36,40) reported that women's who have relatively better education were less likely to have unintended pregnancy ~~as~~ compared to those who didn't have formal education. It is reasonable that as educational level increases ~~the~~ awareness on the reproductive health also increases. Additionally as educational level increases the spousal communication on pregnancy, awareness of long-term family planning and receiving adequate ANC also increases. However, in two studies women's who have relatively better education were more likely to have unintended pregnancy (31,39).
Moreover, in this systematic review and meta-analysis, we observed that as time elapse to reach the nearest health facility for providing contraceptive increases women's are more likely to have unintended pregnancy (10,29,42). It is reasonable that as the time elapse to reach the nearest health facility increases the probability of missing ANC visit also increases which in turn associated with unintended pregnancy (13,20).

Limitations

This study was not without limitations. Firstly, the review was limited to only articles published in English language. Secondly, all of the included studies were cross-sectional, which limits ~~to~~ assess cause-effect relationship. Thirdly, we were unable to show the pooled odds ratio for all variables associated with unintended pregnancy because the included studies classified the variables in different ways.

Conclusions

In this study, a high prevalence of unintended pregnancy was noticed. Lack of spousal communication, never used family planning, maternal education and household wealth level were significantly associated with unintended pregnancy. This study implies ~~to~~ the need for plans

and policies to improve the awareness of contraceptive utilization and strengthen spousal communication related to pregnancy. Emphasis should be given for those women distant to health facilities, unmarried and teenagers.

Refernces

1. Santelli BJ, Rochat R, Hatfield- K, Gilbert C, Curtis K, Cabral R, et al. The Measurement and Meaning of Unintended Pregnancy. 1999;94–101.
2. Unintended Pregnancy Prevention | Unintended Pregnancy - CDC-(Dec 23, 2016 - https://www.cdc.gov/reproductivehealth/contraception/unintendedpregnancy/index.htm.
3. Bearak J, Popinchalk A, Alkema L, Sedgh G. Global , regional , and subregional trends in unintended pregnancy and its outcomes from 1990 to 2014 : estimates from a Bayesian hierarchical model. Lancet Glob Heal [Internet]. 2014;6(4):e380–9. Available from: http://dx.doi.org/10.1016/S2214-109X(18)30029-9
4. Central Statistical Agency (CSA) [Ethiopia] and ICF. 2016. Ethiopia Demographic and Health Survey 2016. Addis Ababa, Ethiopia, and Rockville, Maryland, USA: CSA and ICF.
5. Singh A, Singh A, Thapa S. Adverse Consequences of Unintended Pregnancy for Maternal and Child Health in Nepal. 2015;
6. Kabir, Sandra M.: Causes and Consequences of Unwanted Pregnancy from Asian Women's Perspectives. International Journal of Obstetrics. 1989. Supple 3 .p.9-14.
7. Mumah, J, Kabiru, CW, Mukiira, C, Brinton, J, Mutua, M, Izugbara, C, Birungi, H. and Askew, I. 2014. "Unintended Pregnancies in Kenya: A Country Profile," STEP UP Research Report. Nairobi: African Population and Health Research Center.
8. Induced Abortion and Postabortion Care in Ethiopia,Ipas,January 2017.
9. Rahman M, Rahman M, Tareque I, Ferdos J. Maternal Pregnancy Intention and Professional Antenatal Care Utilization in Bangladesh : A Nationwide Population-Based Survey. 2016;1–15.
10. Melese KG, Gebrie MH, Badi MB, Mersha WF. Unintended Pregnancy in Ethiopia : Community Based Cross-Sectional Study. 2016;2016.
11. Basinga P, Moore AM, Singh S, Remez L, Birungi F, Nyirazinyoye L. Unintended Pregnancy And Induced Abortion In Rwanda:Causes and Consequences. Guttmacher Inst.

12. Wado YD, Afework MF, Hindin MJ. Effects of Maternal Pregnancy Intention , Depressive Symptoms and Social Support on Risk of Low Birth Weight : A Prospective Study from Southwestern Ethiopia. 2014;9(5):1–7.
13. Wado YD, Afework MF, Hindin MJ. Unintended pregnancies and the use of maternal health services in southwestern Ethiopia. BMC Int Health Hum Rights [Internet]. 2013;13(1):1. Available from: BMC International Health and Human Rights
14. Cochrane Book Series. Cochrane Handbook for Systematic Reviews of Interventions.
15. Hoy D, Brooks P, Woolf A, Blyth F, March L, Bain C, et al. Assessing risk of bias in prevalence studies : modification of an existing tool and evidence of interrater agreement. J Clin Epidemiol. 2012;65(9):934–9.
16. Wang N. How to Conduct a Meta-Analysis of Proportions in R : A Comprehensive Tutorial Conducting Meta-Analyses of Proportions in R. 2018;(June).
17. Higgins JPT, Thompson SG, Deeks JJ, Altman DG. Measuring inconsistency in meta-analyses. :557–60.
18. Abame DE, Abera M, Tesfay A, Yohannes Y, Ermias D, Markos T, et al. Relationship Between Unintended Pregnancy and Antenatal Care Use During Pregnancy in Hadiya Zone, Southern Ethiopia. J Reprod Infertil. 2019;20(1):42–51.
19. Worku S, Fantahun M. Unintended pregnancy and induced abortion in a town with accessible family planning services : The case of Harar in eastern Ethiopia.
20. Sectional C, Abayu H, Birhanu Z, Nega A, Kidanemariam A. Prevalence and Associated Factors of Unintended Pregnancy in Welkaite. 2015;2(2).
21. Admasu E, Mekonnen A, Setegn T, Abeje G. Level of unintended pregnancy among reproductive age women in Bahir Dar city administration , Northwest Ethiopia. BMC Res Notes. 2018;1–5.
22. Fite RO, Mohammedamin A, Abebe TW. Unintended pregnancy and associated factors among pregnant women in Arsi Negele. BMC Res Notes. 2018;1–7.
23. Gite A, Liulseged N, Seyife H, Abrha Y, Workineh Y, Shegaze M, et al. Unintended Pregnancy: Magnitude and Associated Factors among Pregnant Women in Arba Minch Town, Gamo Gofa Zone, Ethiopia, 2015. Reprod Syst Sex Disord Curr Res. 2016;5(4):6.
24. Hamdela B, Abebe G, Tilahun T. Unwanted Pregnancy and Associated Factors among Pregnant Married Women in Hosanna Town , Southern. 2012;7(6).

25. Teshome FT, Hailu AG, Teklehaymanot AN. Prevalence of unintended pregnancy and associated factors among married pregnant women in Ganji woreda , west Wollega Oromia region , Ethiopia. 2014;2(2):92–101.
26. Tsegaye AT, Mengistu M, Shimeka A. Prevalence of unintended pregnancy and associated factors among married women in west Belessa Woreda , Northwest. 2018;1–8.
27. Yenealem F, Niberet G. Prevalence and associated factors of unintended pregnancy among pregnant woman in Gondar town , North west Ethiopia ,. BMC Res Notes. 2019;1–5.
28. Health H, Shewa N. Assessment of the Prevalence of Unplanned Pregnancy and Associated Factors among Pregnant Women Attending Antenatal Care Unit at Journal of Women ' s Health Care. 2017;6(6).
29. Darega B, Dida N. Unplanned Pregnancy: Prevalence and Associated Factors among Antenatal Care Attending Women in Bale Zone, Oromiya Region, Southeast Ethiopia: A Facility - based Cross Sectional Study. 2015;15(4).
30. Liyew B, Desalegn B, Ayalew Y. Prevalence of Unintended Pregnancy and Associated Factors among Currently Pregnant Mothers in Kombolcha, Southern Wollo, Ethiopia, 2017.
31. Feyisso M, Girma A, Yimam H, Hailu S. Journal of Women ' s Health Care Magnitude of Unintended Pregnancy and its Determinants among Pregnant Women Visiting Dilla University Referral Hospital , South. 2017;6(4).
32. Gebreamlak W, Aragaw A, Lemma S, Demilew W. Magnitude and factors influencing unintended pregnancy among pregnant women attending antenatal care at Felege Hiwot referral hospital , Northwest Ethiopia : A cross-sectional study. 2014;2(4):261–9.
33. Gizaw W, Fetene G, Mohammed S, Alemu A. iMedPub Journals Magnitude of Unplanned Pregnancy and its Associated Factors among Pregnant Women Attending Antenatal Care at Tepi General Hospital Sheka. 2018;1–8.
34. Goshu YA, Yitayew AE (2019) Prevalence and determinant factors of unintended pregnancy among pregnant women attending antenatal clinics of Addis Zemen hospital. PLoS ONE 14(1): e0210206. https://doi.org/10.1371/journal. pone.0210206.
35. KAHASAY S, SHEMELIS T. ASSESMENT OF MAGNITUDE AND FACTORS ASSOCIATED WITH UNINTENDED PREGNANCY AMONG PREPARATORY

HIGHSCHOOL STUDENTS,ADDIS ABABA,ETHIOPIA. 2015;
36. Kassie T, Geteneh M, Ali A, Tefera W. Magnitude and factors associated with unintended pregnancy among pregnant women in Addis Ababa, Ethiopia. Glob J Med PUBLIC Heal. 2017;6(4):15.
37. Kibret A, Bayu H, Merga M. Prevalence of Unintended Pregnancy and Associated Factors among Pregnant Women Attending Antenatal Clinics in Debre-markos Town, North West Ethiopia 2012. Women's Heal Care. 2014;4(3).
38. Mulat S, Fekadu M, Abera H, Bekele G, Bedaso A. Journal of Women ' s Health Care Prevalence of Unplanned Pregnancy and Associated Factors among Mothers Attending Antenatal Care at Hawassa City Public Hospitals , Hawassa , SNNPR ,. 2017;6(4).
39. Habte D, Teklu S, Melese T, Magafu MGMD. Correlates of Unintended Pregnancy in Ethiopia : Results From a National Survey. 2013;8(12):1–8.
40. Tebekaw Y, Aemro B, Teller C. Prevalence and determinants of unintended childbirth in Ethiopia. 2014;1–9.
41. Abayu H, Birhanu Z, Nega A, Kidanemariam3 A. Prevalence and Associated Factors of Unintended Pregnancy in Welkaite Woreda, Tigray and North Ethiopia Cross Sectional Study by 2012. J Pregnancy Child Heal. 2015;2(2).
42. Kassa N, Berhane Y, Worku A. Predictors of unintended pregnancy in Kersa ,. 2012;1–7.
43. Yakubu I, Salisu WJ. Determinants of adolescent pregnancy in sub-Saharan Africa: a systematic review. BMC Reprod Heal. 2018;
44. Mohamed EAB, Hamed AF, Yousef FMA, Ahmed EA. Prevalence , determinants , and outcomes of unintended pregnancy in Sohag district , Egypt. 2019;9.
45. Ameyaw EK. Prevalence and correlates of unintended pregnancy in Ghana: Analysis of 2014 Ghana Demographic and Health Survey. Matern Heal Neonatol Perinatol. 2018;4(1):1–6.
46. Palamuleni ME, Adebowale AS. Prevalence and determinants of unintended pregnancies in Malawi. African Population Studies. 2014 Apr 29;28(1):551-63.
47. Adeniyi OV, Ajayi AI, Moyaki MG, Goon D Ter, Avramovic G, Lambert J. High rate of unplanned pregnancy in the context of integrated family planning and HIV care services in South Africa. BMC Health Serv Res. 2018;18(1):4–11.
48. Hall JA, Barrett G, Phiri T, Copas A, Malata A, Stephenson J (2016) Prevalence and

Determinants of Unintended Pregnancy in Mchinji District, Malawi; Using a Conceptual Hierarchy to Inform Analysis. PLoS ONE 11(10): e0165621. doi:10.1371/journal.pone.01656.

49. Ndziessi G, Bitemo M, Kaboru BB. Unintended Pregnancies and Associated Factors among Contraceptive Users: a Study from Referral Hospitals in Brazzaville, Republic of Congo. SM J Community Med. 2016;2(1):1015.

50. Metcalfe A, Talavlikar R, Du Prey B, Tough SC. Exploring the relationship between socioeconomic factors, method of contraception and unintended pregnancy. Reprod Health [Internet]. 2016;13(1):1–8. Available from: http://dx.doi.org/10.1186/s12978-016-0151-y

51. Omani-Samani R, Amini Rarani M, Sepidarkish M, Khedmati Morasae E, Maroufizadeh S, Almasi-Hashiani A. Socioeconomic inequality of unintended pregnancy in the Iranian population: A decomposition approach. BMC Public Health. 2018;18(1):1–8.

52. Roy TK, Singh BP (2016) Correlates of Unwanted Births in Bangladesh: A Study through Path Analysis. PLoS ONE 11(10): e0164007. https://doi.org/10.1371/journal.pone.0164007.

53. Africa S, Haffejee F, Connor LO, Govender N, Reddy P, Sibiya MN, et al. Factors associated with unintended pregnancy among women attending a public health facility in Factors associated with unintended pregnancy among women attending a public health facility in KwaZulu-Natal , South Africa. South African Fam Pract [Internet]. 2018;6190:1–5. Available from: http://doi.org/10.1080/20786190.2017.1396790

54. Ikamari L, Izugbara C, Ochako R. Prevalence and determinants of unintended pregnancy among women in Nairobi , Kenya. 2013;1–9.

55. Finer LB et al. Contraception. 2011;84:478–485.

Click here to download Figure Fig 1.docx

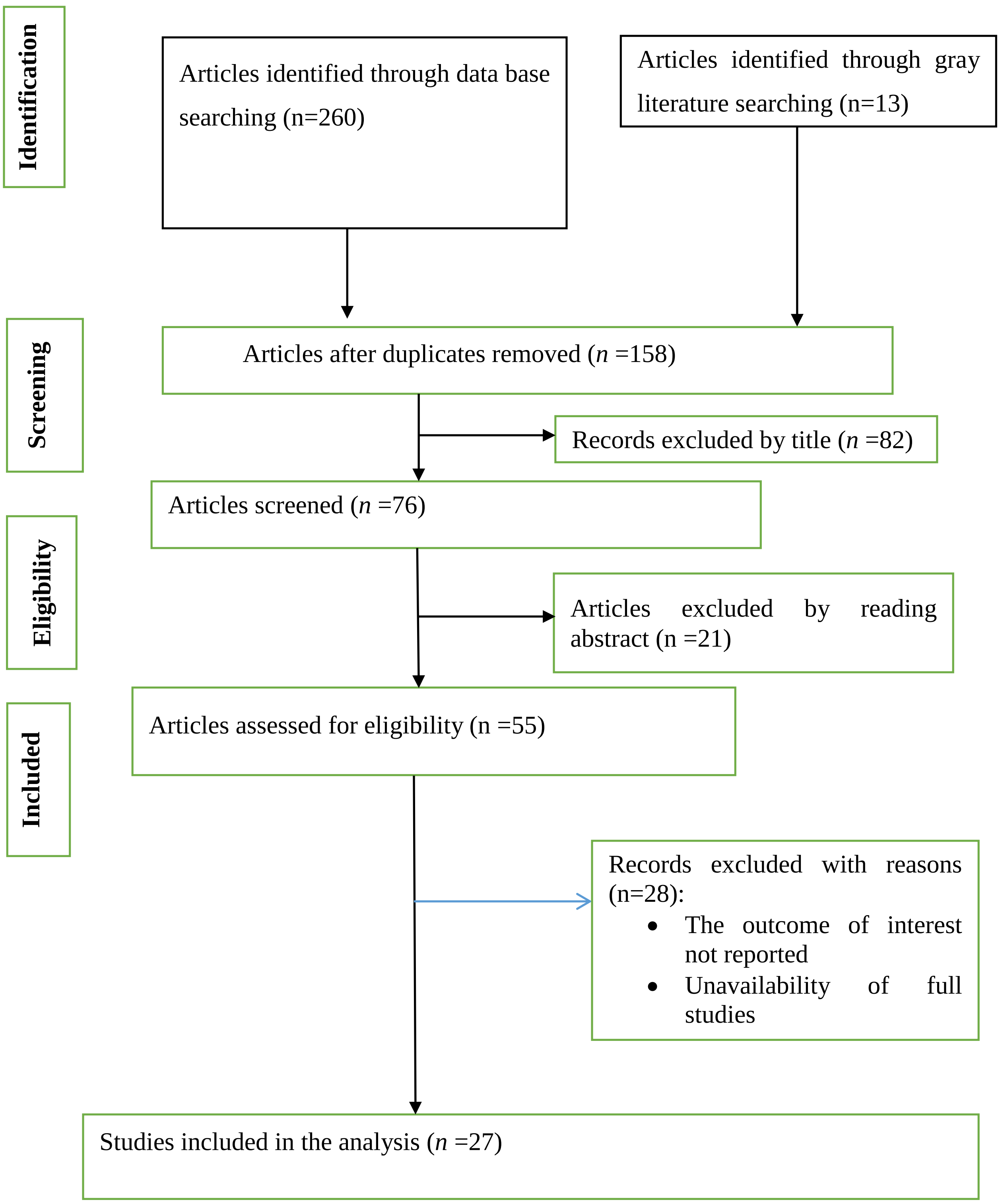

Figure 1: Flow chart diagram describing the selection of studies included in the systematic review and meta-analysis of prevalence and determinants of unintended pregnancy in Ethiopia.

Figure
Click here to download Figure Fig 2.docx

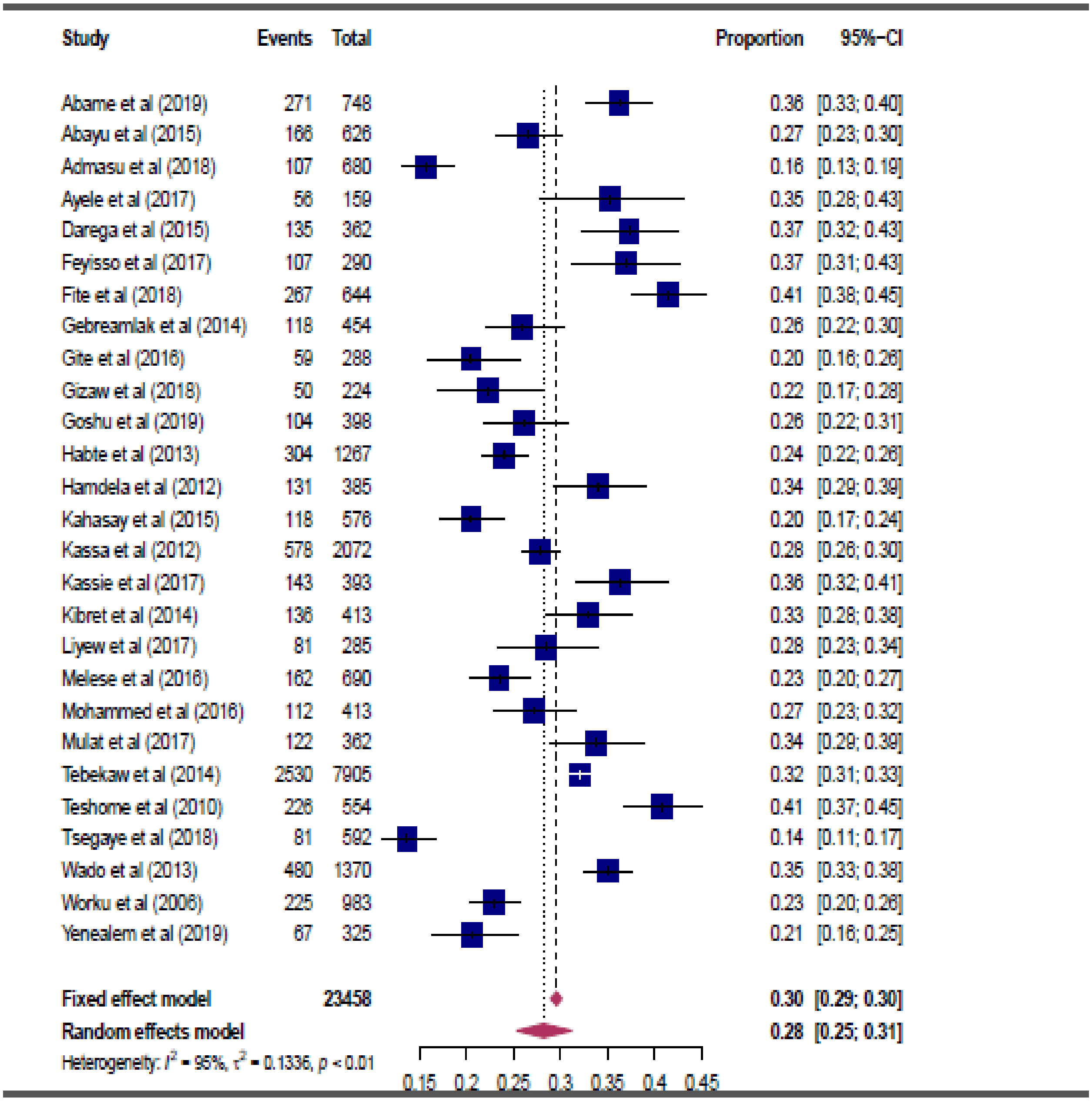

Fig 2. Forest plot of the pooled prevalence of unintended pregnancy in Ethiopia

Figure Click here to download Figure Fig 3.docx

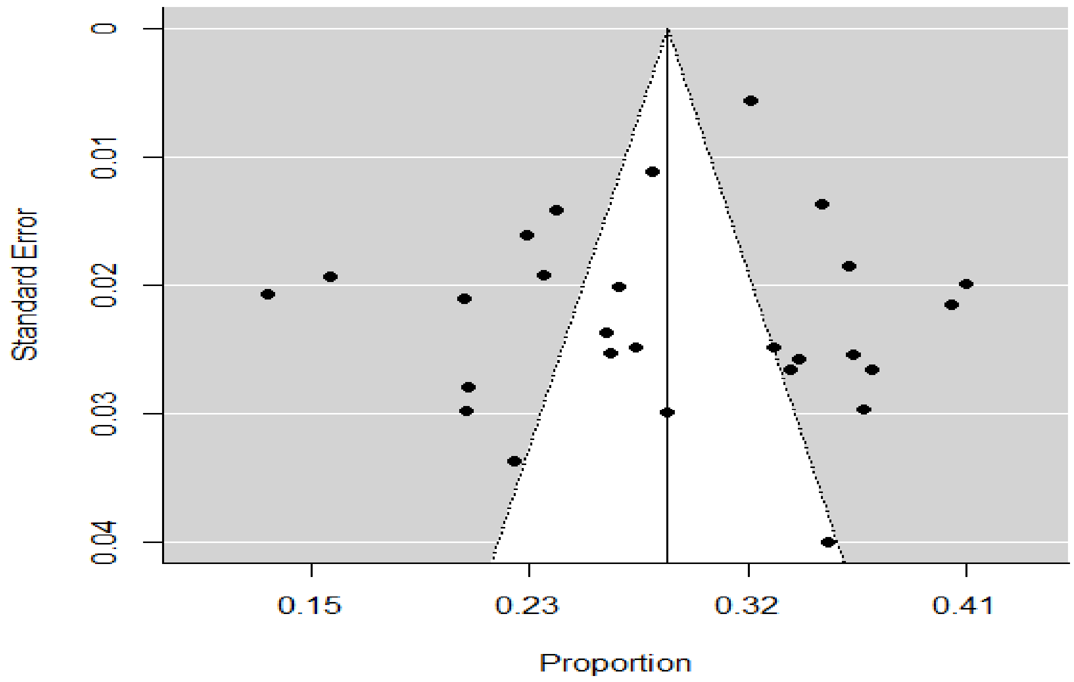

Fig 3: Funnel plot of the prevalence of unintended pregnancy in Ethiopia

Figure Click here to download Figure Fig 4.docx

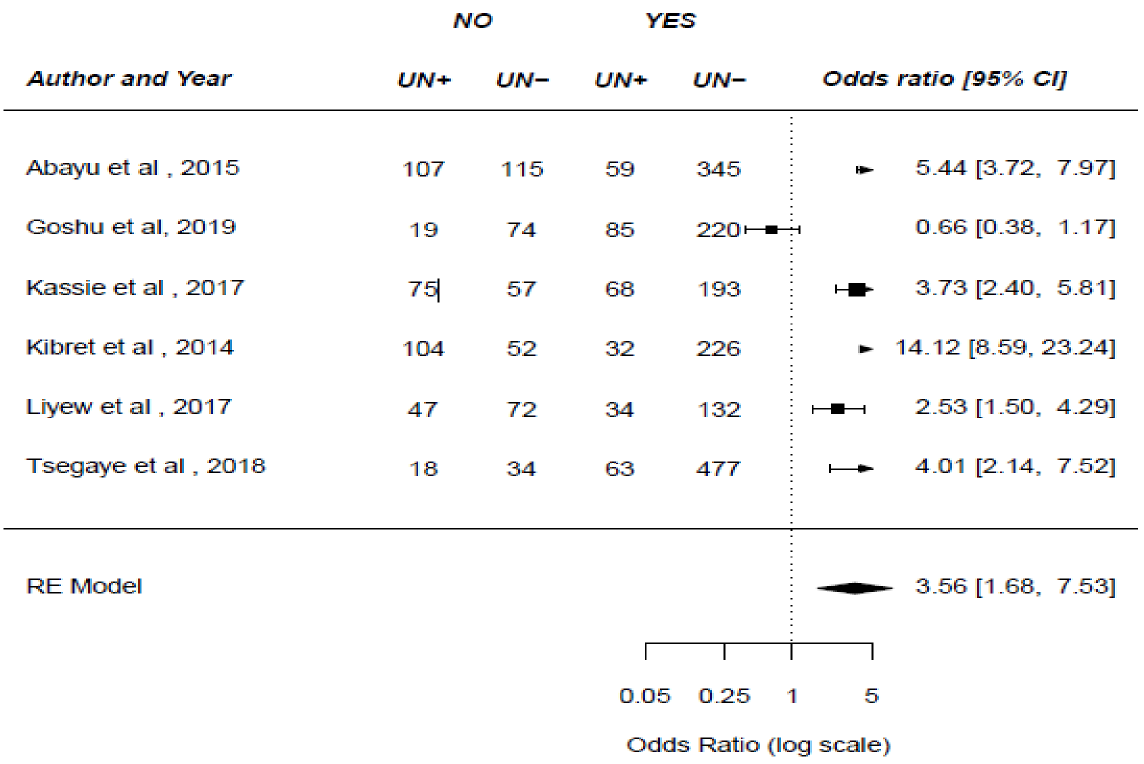

Fig 4. The pooled odds ratio of the association between spousal communication about family planning and unintended pregnancy in Ethiopia.

Figure Click here to download Figure Fig 5.docx

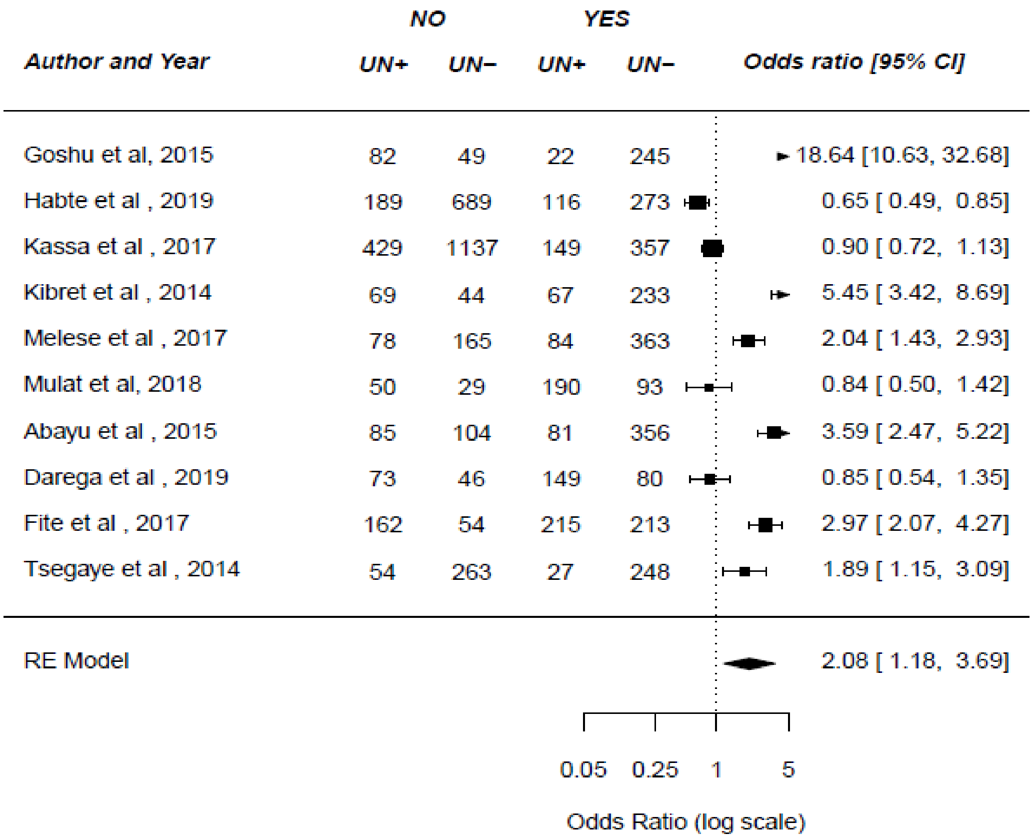

Fig 5. The pooled odds ratio of the association between the use of family planning and unintended pregnancy in Ethiopia.

Click here to access/download
Supporting Information
Table S1.xlsx

Click here to access/download
Supporting Information
Table S2.docx

Click here to access/download
Supporting Information
Prisma checklist.doc
