# Supplementary material for: Prevalence and determinants of unintended pregnancy in Ethiopia: A systematic review and meta-analysis of observational studies
Source: PLoS One. 2020 Apr 7;15(4):e0231012. doi: 10.1371/journal.pone.0231012 (PMC7138300; doi:10.1371/journal.pone.0231012)
Supplement: Attachment — Submitted filename: PONE-D-19-21662_R1_reviewer (2).pdf [file pone.0231012.s007.pdf]

PLOS ONE

# Prevalence and determinants of unintended pregnancy in Ethiopia: a systematic review and meta-analysis of observational studies

--Manuscript Draft--

| | |
|---|---|
| Manuscript Number: | PONE-D-19-21662R1 |
| Article Type: | Research Article |
| Full Title: | Prevalence and determinants of unintended pregnancy in Ethiopia: a systematic review and meta-analysis of observational studies |
| Short Title: | Unintended pregnancy in Ethiopia |
| Corresponding Author: | Muluneh Alene Addis, MPH<br>Debre Markos University<br>Debre Markos, ETHIOPIA |
| Keywords: | Prevalence, Unplanned pregnancy, Ethiopia, Meta-analysis, Maternal morbidity and mortality |
| Abstract: | Abstract<br>Background: Unintended pregnancy has significant consequences for the health and welfare of women and children. Despite this, a number of studies with inconsistent findings were conducted to reduce unintended pregnancy in Ethiopia; unavailability of a nationwide study that determines the prevalence of unintended pregnancy and its determinants is an important research gap. Thus, this study was conducted to determine the overall prevalence of unintended pregnancy and its determinants in Ethiopia.<br>Methods: We searched from Google Scholar, PubMed, Science Direct, Web of Science, CINAHL, and Cochrane Library databases for studies. Each of the original studies was assessed using a tool for the risk of bias of observational studies. The heterogeneity of studies was also assessed using I2 test statistics. Data were pooled and a random effect meta-analysis model was fitted to provide the overall prevalence of unintended pregnancy and its determinants in Ethiopia. In addition, the subgroup analyses were performed to investigate how the prevalence of unintended pregnancy varies across different groups of studies.<br>Results: Twenty-seven studies that satisfy the eligibility criteria were included. We found that the overall prevalence of unintended pregnancy in Ethiopia was 28% (95% CI: 25-31). The subgroup analysis showed that the highest prevalence of unintended pregnancy was observed from the Oromiya region (33.8%) followed by Southern Nations, Nationalities, and Peoples' region (30.6%), and the lowest was in Harar. In addition, the pooled prevalence of unintended pregnancy was 26.4% (20.8-32.4) and 30.0% (26.6-33.6) for community-based cross-sectional and institution-based cross-sectional studies respectively. The pooled analysis showed that not communicating with one's husband about family planning was more likely to lead to unintended pregnancy (OR: 3.56, 95%CI: 1.68-7.53). The pooled odds ratio also showed that unintended pregnancy is more likely among women who never use family planning methods (OR: 2.08, 95%CI: 1.18-3.69). Furthermore, the narrative review of this study showed that maternal education, age, and household wealth index are strongly associated with an unintended pregnancy.<br>Conclusions: In this study, the prevalence of unintended pregnancy was high. Lack of spousal communication, never using family planning, maternal education, and household wealth level were significantly associated with an unintended pregnancy. This study implies the need to develop plans and policies to improve the awareness of contraceptive utilization and strengthen spousal communication related to pregnancy.<br>Key words: Prevalence, Unplanned pregnancy, Ethiopia, Meta-analysis, Maternal morbidity and mortality |
| Order of Authors: | Muluneh Alene Addis, MPH |
| | Leltework Yismaw Wondemagegn |
| | Yebelay Berelie Berhan |
| | Bekalu Kassie Alemu |
| | |

Powered by Editorial Manager® and ProduXion Manager® from Aries Systems Corporation

| | |
|---|---|
| | Reta Yeshamble Kessete |
| | Moges Agazhe Assemie |
| **Response to Reviewers:** | Author's response to reviews<br><br>Title: Prevalence and determinants of unintended pregnancy in Ethiopia: a systematic review and meta-analysis of observational studies<br><br><br>Authors<br>Author's email addresses<br>MA: mulunehadis@gmail.com<br>LY: lielt.yismaw@gmail.com<br>YB: yebelay.ma@gmail.com<br>BK: bekalukassiedmu@gmail.com<br>RY: reta.yeshambel@gmail.com<br>MAA: agazhemoges@gmail.com<br><br>Date: 17 October 2019<br>Dear Editor,<br>We thank you for the chance to resubmit our revised manuscript. Also, we would like to thank the reviewers for sharing the view and experience. The comments are very important that will improve the manuscript. The point-by-point responses for each of the comments are provided in the following pages. We hope that the revisions meet your standards and that the paper would be published in your journal. We look forward to working with you towards a final published product.<br><br>Sincerely,<br>Muluneh Alene, MPH<br>On behalf of co-authors<br><br><br><br><br>Point by point responses to queries<br>Reviewer#1<br>Comments and points raisedAuthors  response<br>1."Delete first sentence of abstract?"Answer: thank you dear reviewer for your constructive comments!<br>oBased on the suggestion given, we make modification on the first sentence of the abstract section in the revised form of the manuscript.<br>2."The quality of the English needs major work."<br><br>Answer: Thank you!<br>oThe issue of English language is resolved using English language professionals in the revised form of the manuscript.<br><br>Reviewer#2<br>Comments and points raisedAuthors  response<br>1."Was a time period determined for the search? If yes, what was it? If no, then please mention that."<br>Answer: thank you dear reviewer for your constructive comments!<br>oThe searching time period was already stated and it was between the 15th of February and 30th of April 2019<br>2."How did you search for unpublished studies?"<br><br>Answer: Thank you!<br>oTo find unpublished papers relevant to this study, some research centers including Addis Ababa digital library were searched.<br>3."Was any attempt made to contact authors of studies for which full papers were not obtained?"<br>Answer: Thank you!<br>oWe were planned to contact the primary author by email at least three times if a study is not fully available. But, studies searched for this systematic review and meta- |

analysis were fully obtained.
4.“The writing needs quite a bit of improvement in terms of flow.”Answer: Thank you!
oBased on your suggestion, we revised the flow of idea in the revised form of the manuscript and we make highlight for changes.

Reviewer#3
Comments and points raisedAuthors response
1.“Literature search: it is limited to 2 data bases and Google scholar, which is not comprehensive. The authors should have searched more relevant databases. They mentioned that they searched national databases, however, there is no description of such database. The search was limited to English language. As this study is investigating the prevalence of unintended pregnancy in a specific county/ region, it must have been included the articles in local language. This may increase the risk of reporting and publication bias.”
Answer: thank you dear reviewer for your constructive comments!
oBased on the comments given and to make comprehensive, in the revised form of the manuscript, we extend our searches for studies from PubMed/MEDLINE, Web of Science, CINAHL, Google Scholar, Science Direct and Cochrane Library.
oWe were searched the “Ethiopian Journal of Public Health and Nutrition” for the national databases.
oYou are right dear reviewer; not including articles published in local language may increase the risk of reporting and publication bias.
o But, we were searched for articles published in local language related to this study, but we can’t get such articles.
2.“Authors should explain the reasons in heterogeneity among studies. Why it exists, and how it affects the credibility of the overall results?”

Answer: Thank you!
oBased on the comment given, we included the possible reasons for heterogeneity among included studies.
oIn this systematic review and meta-analysis, the possible sources of heterogeneity among studies are differences in study participants, study design, risk of bias and data collection period.
oTo account the heterogeneity among the included studies, subgroup analysis and a random effect meta-analysis with an estimation of DerSimonian and Laird method was performed.
3.“Discussion: there is no mention of women’s age, time to reach the nearest health facility, household wealth, being married… in results section, but they show up in dissuasion as the potential determinants on the prevalence of unintended pregnancies”
oBased on the comments given, we included the stated variables in the result section.

Reviewer#4
Comments and points raisedAuthors response
1.“Parity, occupation, religion and economic status are important determinants of unintended pregnancy. Could the authors provide insight into why they were not significantly associated with unintended pregnancy in the review?”Answer: thank you dear reviewer for your constructive comments!
oIn the revised form of the manuscript, we already considered the association between the mentioned variables and unintended pregenancy.
2.“As readers may not be familiar with Ethiopia, please define SNNPR at first instance.”

Answer: Thank you!
oBased on the comment given, we already define the word “SNNPR” at first instance in the revised form of the manuscript.

Reviewer#5
Comments and points raisedAuthors response
1.“There are 195 English language corrections that I strongly suggest you follow in the Comments section of the attached PDF file.”Answer: thank you dear reviewer for your constructive comments!
oWe thank you for your revision, and we considered all English language corrections in the revised form of the manuscript.

| | |
|---|---|
| | Thank you!!! |
| **Additional Information:** | |
| **Question** | **Response** |
| **Financial Disclosure**<br><br>Enter a financial disclosure statement that describes the sources of funding for the work included in this submission. Review the submission guidelines for detailed requirements. View published research articles from *PLOS ONE* for specific examples.<br><br>This statement is required for submission and **will appear in the published article** if the submission is accepted. Please make sure it is accurate.<br><br>**Unfunded studies**<br>Enter: *The author(s) received no specific funding for this work.*<br><br>**Funded studies**<br>Enter a statement with the following details:<br>• Initials of the authors who received each award<br>• Grant numbers awarded to each author<br>• The full name of each funder<br>• URL of each funder website<br>• Did the sponsors or funders play any role in the study design, data collection and analysis, decision to publish, or preparation of the manuscript?<br>• **NO** - Include this sentence at the end of your statement: *The funders had no role in study design, data collection and analysis, decision to publish, or preparation of the manuscript.*<br>• **YES** - Specify the role(s) played.<br><br>* typeset | The author(s) received no specific funding for this work. |
| **Competing Interests**<br><br>Use the instructions below to enter a competing interest statement for this submission. On behalf of all authors, disclose any competing interests that could be perceived to bias this work—acknowledging all financial support and any other relevant financial or non- | The authors have declared that no competing interests exist. |

| | |
|---|---|
| financial competing interests.<br><br>This statement **will appear in the published article** if the submission is accepted. Please make sure it is accurate. View published research articles from *PLOS ONE* for specific examples.<br><br>**NO authors have competing interests**<br><br>Enter: *The authors have declared that no competing interests exist.*<br><br>**Authors with competing interests**<br><br>Enter competing interest details beginning with this statement:<br><br>*I have read the journal's policy and the authors of this manuscript have the following competing interests: [insert competing interests here]*<br><br>* typeset | |
| **Ethics Statement**<br><br>Enter an ethics statement for this submission. This statement is required if the study involved:<br><br>• Human participants<br>• Human specimens or tissue<br>• Vertebrate animals or cephalopods<br>• Vertebrate embryos or tissues<br>• Field research<br><br>Write "N/A" if the submission does not require an ethics statement.<br><br>General guidance is provided below. Consult the submission guidelines for detailed instructions. **Make sure that all information entered here is included in the Methods section of the manuscript.** | N/A |

Powered by Editorial Manager® and ProduXion Manager® from Aries Systems Corporation

| | |
|---|---|
| **Format for specific study types**<br><br>**Human Subject Research (involving human participants and/or tissue)**<br>• Give the name of the institutional review board or ethics committee that approved the study<br>• Include the approval number and/or a statement indicating approval of this research<br>• Indicate the form of consent obtained (written/oral) or the reason that consent was not obtained (e.g. the data were analyzed anonymously)<br><br>**Animal Research (involving vertebrate animals, embryos or tissues)**<br>• Provide the name of the Institutional Animal Care and Use Committee (IACUC) or other relevant ethics board that reviewed the study protocol, and indicate whether they approved this research or granted a formal waiver of ethical approval<br>• Include an approval number if one was obtained<br>• If the study involved *non-human primates*, add *additional details* about animal welfare and steps taken to ameliorate suffering<br>• If anesthesia, euthanasia, or any kind of animal sacrifice is part of the study, include briefly which substances and/or methods were applied<br><br>**Field Research**<br><br>Include the following details if this study involves the collection of plant, animal, or other materials from a natural setting:<br>• Field permit number<br>• Name of the institution or relevant body that granted permission | |
| **Data Availability**<br><br>Authors are required to make all data underlying the findings described fully available, without restriction, and from the time of publication. PLOS allows rare exceptions to address legal and ethical concerns. See the PLOS Data Policy and FAQ for detailed information. | Yes - all data are fully available without restriction |

| | |
|---|---|
| A Data Availability Statement describing where the data can be found is required at submission. Your answers to this question constitute the Data Availability Statement and **will be published in the article**, if accepted.<br><br>**Important:** Stating ‘data available on request from the author’ is not sufficient. If your data are only available upon request, select ‘No’ for the first question and explain your exceptional situation in the text box.<br><br>Do the authors confirm that all data underlying the findings described in their manuscript are fully available without restriction? | |
| **Describe where the data may be found in full sentences. If you are copying our sample text, replace any instances of XXX with the appropriate details.**<br><br>• If the data are **held or will be held in a public repository**, include URLs, accession numbers or DOIs. If this information will only be available after acceptance, indicate this by ticking the box below. For example: *All XXX files are available from the XXX database (accession number(s) XXX, XXX.)*.<br>• If the data are all contained **within the manuscript and/or Supporting Information files**, enter the following: *All relevant data are within the manuscript and its Supporting Information files.*<br>• If neither of these applies but you are able to provide **details of access elsewhere**, with or without limitations, please do so. For example:<br><br>*Data cannot be shared publicly because of [XXX]. Data are available from the XXX Institutional Data Access / Ethics Committee (contact via XXX) for researchers who meet the criteria for access to confidential data.*<br><br>*The data underlying the results presented in the study are available from (include the name of the third party* | All relevant data are within the manuscript and its Supporting Information files. |

| | |
|---|---|
| *and contact information or URL).*<br>• This text is appropriate if the data are owned by a third party and authors do not have permission to share the data.<br><br>* typeset | |
| Additional data availability information: | |

Manuscript

Click here to access/download;Manuscript;Manuscript.docx

# Prevalence and determinants of unintended pregnancy in Ethiopia: a systematic review and meta-analysis of observational studies

Muluneh Alene[1*], Leltework Yismaw[1], Yebelay Berelie[2], Bekalu Kassie[3], Reta Yeshambel[4], Moges Agazhe Assemie[1]

1Department of Public Health, Debre Markos University, Debre Markos, Ethiopia
2Department of Statistics, Debre Markos University, Debre Markos, Ethiopia
3Department of Midwifery, Debre Markos University, Debre Markos, Ethiopia
4Department of Biology, Mizan-Tepi University, Teppi, Ethiopia

**Author's email addresses**
MA: mulunehadis@gmail.com
LY: lielt.yismaw@gmail.com
YB: yebelay.ma@gmail.com
BK: bekalukassiedmu@gmail.com
RY: reta.yeshambel@gmail.com
MAA: agazhemoges@gmail.com

**[*]Corresponding author**
mulunehadis@gmail.com

**Abstract**

**Background:** Unintended pregnancy has significant consequences for the health and welfare of women and children. Despite this, a number of studies with inconsistent findings were conducted to reduce unintended pregnancy in Ethiopia; unavailability of a nationwide study that determines the prevalence of unintended pregnancy and its determinants is an important research gap. Thus, this study was conducted to determine the overall prevalence of unintended pregnancy and its determinants in Ethiopia**.**

**Methods:** We searched from Google Scholar, PubMed, Science Direct, Web of Science, CINAHL, and Cochrane Library databases for studies. Each of the original studies was assessed using a tool for the risk of bias of observational studies. The heterogeneity of studies was also assessed using $I^2$ test statistics. Data were pooled and a random effect meta-analysis model was fitted to provide the overall prevalence of unintended pregnancy and its determinants in Ethiopia. In addition, the subgroup analyses were performed to investigate how the prevalence of unintended pregnancy varies across different groups of studies.

**Results:** Twenty-seven studies that satisfy the eligibility criteria were included. We found that the overall prevalence of unintended pregnancy in Ethiopia was 28% (95% CI: 25-31). The subgroup analysis showed that the highest prevalence of unintended pregnancy was observed from the Oromiya region (33.8%) followed by Southern Nations, Nationalities, and Peoples' region (30.6%), and the lowest was in Harar. In addition, the pooled prevalence of unintended pregnancy was 26.4% (20.8-32.4) and 30.0% (26.6-33.6) for community-based cross-sectional and institution-based cross-sectional studies respectively. The pooled analysis showed that not communicating with one's husband about family planning was more likely to lead to unintended pregnancy (OR: 3.56, 95%CI: 1.68-7.53). The pooled odds ratio also showed that unintended pregnancy is more likely among women who never use family planning methods (OR: 2.08, 95%CI: 1.18-3.69). Furthermore, the narrative review of this study showed that maternal education, age, and household wealth index are strongly associated with an unintended pregnancy.

**Conclusions:** In this study, the prevalence of unintended pregnancy was high. Lack of spousal communication, never using family planning, maternal education, and household wealth level

were significantly associated with an unintended pregnancy. This study implies the need to develop plans and policies to improve the awareness of contraceptive utilization and strengthen spousal communication related to pregnancy.

**Key words:** Prevalence, Unplanned pregnancy, Ethiopia, Meta-analysis, Maternal morbidity and mortality

## Introduction

Unintended pregnancy is a pregnancy which is either mistimed or unwanted (1,2). It is a public health problem and a risk factor for adverse health outcomes, particularly for maternal and child health (3). Though the rate of unintended pregnancy fell worldwide between 1990 and 2014, it dropped less sharply in developing regions than in developed regions (3). Ethiopia is one of the developing countries with a high prevalence of unintended pregnancy. In Ethiopia, more-than one-third (38%) of pregnancies were unintended in 2014; slightly lower than in 2008 which was 42%. According to results from the 2016 Ethiopian Demographic and Health Survey (EDHS) of all births in the past five years and current pregnancies, 25% are unintended. Also, the 2016 EDHS report showed that the overall difference between the wanted fertility rate and the total fertility rate is one child, which suggests that Ethiopian women are currently having, on average, one child more than they want (4).

Pregnancies should be planned before conception unless a woman may not be in optimal health for childbearing (2). Unintended pregnancy leads to maternal mortality and morbidity due to the complications of unsafe abortion, miscarriage, and unplanned births, which burdens the health system at all (5–7). Annually, more than 1 in 10 pregnancies end in abortion, and 1 in 27 mothers die due to the complications of pregnancy or childbirth in Ethiopia (8). A woman with an unintended pregnancy will have low physical and mental health, low self-care, and depression during pregnancy. These lead to poor Antenatal Care (ANC) service utilization and postpartum depression, which is a risk factor for unfavorable pregnancy outcome, and maternal morbidity and mortality (7,9). Consequently, the newborns of unintended pregnancies are faced with low birth weight and inadequate vaccinations which increases the risk for childhood illnesses (5,7,10–12). Previous evidence showed that unintended pregnancy mainly results from inconsistent or incorrect use of contraceptive methods, and women’s are less likely than men to want more children no matter how many children they already have (2,13).

In Ethiopia, a number of studies were conducted to estimate the magnitude and to identify the determinants of unintended pregnancy. However, the reported prevalence and determinants in these fragmented studies vary depending on the characteristics of study participants, the type of design employed and the variables analyzed. Combined findings of existing studies significantly strengthen the quality of evidence investigating the national prevalence and determinants of unintended pregnancy. Thus, this systematic review and meta-analysis was conducted to determine the overall prevalence and determinants of unintended pregnancy in Ethiopia. This review is intended to bring an improvement in the design of future studies related to unintended pregnancy. The findings of this study are also intended to improve health worker's intervention in the area of reproductive health.

## Materials and methods

### Study design and setting

A systematic review and meta-analysis, which aimed to estimate the overall prevalence of unintended pregnancy and its determinants was conducted in Ethiopia. Ethiopia is situated in the horn of Africa, and bordered by Eritrea to the north, Sudan and South Sudan to the west, Kenya to the south, and Djibouti and Somalia to the east. Nearly eight in ten women (78%) live in rural areas in Ethiopia, and half of women age 15-49 (48%) have no education (4).

### Eligibility criteria

In this systematic review and meta-analysis, studies were included with the following criteria: 1) only studies conducted in Ethiopia. 2) Only studies reported in English language, 3) only studies involving pregnant women or had given birth at least once preceding the survey and 4) all observational studies reporting the prevalence and determinants of unintended pregnancy. Both published and unpublished articles were included. Studies which were not fully accessible were excluded because of the inability to assess the quality of studies without their full text.

### Searching for studies

A comprehensive search strategy was done by three (MA, LY, and RY) of the authors. Both published and unpublished articles on unintended pregnancy were searched from international (Google Scholar, PubMed, Science Direct, Web of Science, CINAHL, and Cochrane Library), and national (Ethiopian Journal of Public Health and Nutrition) electronic databases. First,

articles were searched by examining the full titles ("Prevalence and determinants of unintended pregnancy in Ethiopia") and then keywords (unintended pregnancy, unplanned pregnancy, unwanted pregnancy, mistimed pregnancy, determinants, risk factors, associated factors, Ethiopia). These keywords were used separately and in combination using Boolean operators "OR" or "AND". In addition, we searched from the reference lists of all the included studies (snowball technique) to identify any other studies that may have been missed by our search strategy. Finally, all studies were imported into reference management software (Mendeley Desktop).

**Outcome measures and data extraction**

Unintended pregnancy, which is either mistimed or unwanted, was the primary outcome of the study. Unwanted pregnancy occurred when a woman did not want to have any more pregnancies, whereas mistimed pregnancy is a pregnancy that was wanted by the woman at some time, but which occurred sooner than they wanted. The pregnancy intention for the included studies was assessed by interviewing current pregnant women or women who had given birth at least once preceding the survey.

All essential data from the included studies were extracted independently by two (MA and LY) of the authors using a predesigned data abstraction form. This form includes the last name of the first author, publication year, data collection period, study design, region of the study conducted, study population, sample size, response rate, and the magnitude of unintended pregnancy. In addition, we extracted the adjusted odds ratios with corresponding 95% confidence intervals to measure the strength of effects. Deviation in a data extraction process was resolved by discussion and consensus involving all authors.

**Quality assessment for studies**

The quality of meta-analysis depends on the included studies (14). Two authors (MA and YB) assessed the risk of bias for the included studies using the tool of risk of bias assessment for observational studies (15). This tool includes 10 items. The first four items assess the external validity, while the other six items evaluate the internal validity of the study. All items of the tool were filled in for each included study and categorized as low risk bias (if the response is "yes"), higher risk (if the response is "no"), and not clear. The quality of the study was determined by summing the score given for each item. Lastly, unclear risk of bias was categorized as high risk

of bias then; the summary assessment risk of bias for each study was categorized according to the number of high risk of bias: low (≥2), moderate (3-4), and high (≥5).

## Data processing and analysis

After extracting all relevant data using Microsoft excel software, data were exported to R statistical software for meta-analysis. The double arcsine transformation which stabilizes the sampling variance was applied to estimate the weighted average prevalence, and the transformed summary prevalence are converted back for ease of interpretation (16).

We assessed the consistency of studies using $I^2$ test statistics (17). This test examines the null hypothesis that all the included studies are evaluating the same effect. Consequently, since there was heterogeneity between the original studies ($I^2$=96%, p<0.01) a random effect model is needed, and to account for between-study variance a random effect meta-analysis with an estimation of DerSimonian and Laird method was performed. The possible sources of heterogeneity among studies might be differences in study participants, study design, risk of bias and data collection period. Furthermore, subgroup analyses were conducted to investigate how the prevalence of unintended pregnancy varied across different subgroups of studies.

# Results

## Search results

Our comprehensive search for studies was between the 15th of February and the 30th of April, 2019. The flow chart diagram that shows our literature search, study selection, and the number of included studies is presented in (**Fig 1**). Initially, a total of 273 articles were identified during our search, and then 115 articles were excluded due to duplication. Finally, 27 studies that satisfied the eligibility criteria were included in this systematic review and meta-analysis.

**Figure 1**: Flow chart diagram describing the selection of studies included in the systematic review and meta-analysis of prevalence and determinants of unintended pregnancy in Ethiopia.

## Description of the included studies

The detail descriptions of the included studies are shown in (**Table 1**). All studies included in this review were conducted between 2006 and 2019. Of all the included studies, eleven (10,18–27) were community-based cross-sectional, while twelve were institutional-based cross-sectional studies (28–38). The data for the two studies were also taken from the 2011 EDHS (39,40). The

majority of respondents of the original studies were pregnant mothers. The number of participants in each study varied from the lowest of 165 (28) to the highest of 7,759 (40). A study with the smallest study participants was an institutional-based cross-sectional study, while the data for the highest study participants were taken from the 2011 EDHS. Furthermore, nine studies were conducted in the Amhara region, six studies in the Oromiya region, six studies in Southern Nations, Nationalities, and Peoples' Region (SNNPR), two studies in Addis Ababa, one study in Harar, and two studies from the national survey. Moreover, the prevalence of unintended pregnancy ranged from 13.7% (26) to 41.5% (22).

**Table 1**: Descriptions of the included studies conducted in Ethiopia on unintended pregnancy.

| **First author (publication year)** | **Study area (Region)** | **Study design** | **Data collection period** | **Study population** | **Sample size** | **Response rate** | **Prevalence of unintended pregnancy** |
|---|---|---|---|---|---|---|---|
| Abame et al (2019) | SNNPR | CBCS | March 13, 2017 to April 13, 2017 | pregnant mothers | 748 | 97% | 36.2% |
| Abayu et al (2015) | Tigray | CBCS | 24/09/2012 to 18/10/2012 | Pregnant women | 626 | 96.3% | 26% |
| Admasu et al (2018) | Amhara | CBCS | NR | pregnant women or with under 1 child age | 680 | 91% | 15.8% |
| Ayele et al (2017) | Oromiya | IBCS | June 10, 2017 to July 24, 2017 | pregnant women following ANC | 165 | 96% | 35.2% |
| Darega et al (2015) | Oromiya | IBCS | May ,2014 | Women following ANC | 362 | 100% | 37.3% |
| Feyisso et al (2017) | SNNPR | IBCS | February to June, 2016 | Women following | 290 | NR | 36.9% |

| | | | | | | | |
|---|---|---|---|---|---|---|---|
| | | | | ante natal and post natal ANC | | | |
| Fite et al (2018) | Oromiya | CBCS | May 01–July 30, 2017 | Pregnant women | 704 | 91.5% | 41.5% |
| Gebreamlak et al (2014) | Amhara | IBCS | June to July 2012 | Pregnant women | 454 | NR | 26% |
| Gite et al (2016) | SNNPR | CBCS | February 15-March 11,2015 | Pregnant women | 311 | 95.4% | 19.4% |
| Gizaw et al (2018) | SNNPR | IBCS | February 24 to April 24, 2017 | Pregnant women | 224 | 100% | 22.3% |
| Goshu et al (2019) | Amhara | IBCS | April 01 to May 30, 2018 | Pregnant women | 398 | 100% | 26.1% |
| Habte et al (2013) | National level | EDHS | NA | Pregnant women | 1267 | NA | 24% |
| Hamdela et al (2012) | SNNPR | CBCS | April 02 to 15, 2011 | pregnant married women | 385 | 100% | 34% |
| Kahasay et al (2015) | Addis Abeba | IBCS | NR | female students aged from 16-19 years | 576 | 100% | 20.4% |
| Kassa et al | Oromiya | KDSH | December | Pregnant | 2072 | 100% | 27.9% |

| | | | | | | | |
|---|---|---|---|---|---|---|---|
| (2012) | | RC | 2009 to November 2010 | women | | | |
| Kassie et al (2017) | Addis Abeba | IBCS | February to May 2015 | Pregnant women | 393 | 100% | 36.4% |
| Kibret et al (2014) | Amhara | IBCS | April 15 to May 14, 2012 | Pregnant women | 413 | 100% | 32.9% |
| Liyew et al (2017) | Amhara | IBCS | NR | Pregnant women | 285 | 100% | 28.4% |
| Melese et al (2016) | Amhara | CBCS | NR | Pregnant women | 690 | NR | 23.5% |
| Mohammed et al (2016) | Oromiya | IBCS | January 10 to April 13, 2015 | Pregnant women | 413 | 97.9% | 27.1% |
| Mulat et al (2017) | SNNPR | IBCS | NR | Pregnant women | 362 | 100% | 33.7% |
| Tebekaw et al (2014) | National level | EDHS | EDHS, 2011 | Women who had at least one birth | 7,759 | NA | 32% |
| Teshome et al (2010) | Amhara | CBCS | NR | currently married women | 576 | NR | 40.8% |
| Tsegaye et al (2018) | Amhara | CBCS | August to September | Married pregnant | 619 | 95.6% | 13.7% |

| | | | | | | | |
|---|---|---|---|---|---|---|---|
| | | | 2015 | women | | | |
| Wado et al (2013) | Oromiya | HDSS | March,2012 | mothers with alive birth in the two years | 1456 | 94% | 35% |
| Worku et al (2006) | Harar | CBCS | November to December 2001 | Reproductive age women | 983 | 98.3% | 33.3% |
| Yenealem et al (2019) | Amhara | CBCS | April 1–May 30, 2014 | Pregnant women | 325 | 100% | 20.6% |

*CBCS= Community-Based Cross Sectional Study, EDHS= Ethiopia Demographic and Health Survey, HDSS=Health and Demographic Surveillance System, IBCS=Institution-Based Cross Sectional Study, KDS-HRC= Kersa Demographic Surveillance and Health Research Center, NA= Not Applicable, NR=Not Report, SNNRP= Southern Nations, Nationalities, and Peoples' Region*

**Risk of bias assessment for the included studies**

We assessed the risk of bias for each of the original studies using the existing risk-of-bias assessment tool (**Table S1**). Of the total included studies, our summary assessment showed that more-than half (55.6%) of the studies had a low risk of bias, and nearly one-fourth (25.9%) of studies had a moderate risk of bias. Additionally, less than one-fifth (18.5%) of the included studies had a high risk of bias.

**Prevalence of unintended pregnancy in Ethiopia**

In this study, the overall prevalence of unintended pregnancy in Ethiopia was 28% (95% CI: 25-31) (**Fig 2**). We had conducted subgroup analyses to investigate how the prevalence of unintended pregnancy varies across different subgroups of studies (**Table 2**). Consequently, the pooled prevalence of unintended pregnancy was 26.4% (20.8-32.4) and 30.0% (26.6-33.6) for community-based cross-sectional and institution-based cross-sectional studies, respectively. The subgroup analysis based on the region where studies were conducted also showed that the highest prevalence of unintended pregnancy was observed from the Oromiya region [33.8%

(29.0-38.7)] followed by SNNPR [30.6% (25.2-36.2)] and Addis Ababa [28.0% (14.0-44.7)]. The lowest prevalence was also noted in Harar [22.9% (20.3-25.5)]. Furthermore, the pooled prevalence of unintended pregnancy was 28.2% (25.1-31.4) and 28.8% (23.7-34.0) for studies conducted prior and post 2014, respectively.

**Fig 2**. Forest plot of the pooled prevalence of unintended pregnancy in Ethiopia

**Table 2:** Subgroup analysis of studies included in meta-analysis on prevalence and determinants of unintended pregnancy in Ethiopia

| **Subgroup** | **Random effects (95%CI)** | **Test of heterogeneity ($I^2$)** |
|---|---|---|
| **By study design** | | |
| CBCS | 26.4% ( 20.8-32.4) | 96.6% |
| IBCS | 30.0% (26.6-33.6) | 84.4% |
| Overall | 28.2% (24.1-32.5) | 82.5% |
| **By region** | | |
| Amhara | 24.9% (19.2-31.0) | 95.2% |
| Oromiya | 33.8% (29.0-38.7) | 91.2% |
| SNNPR | 30.6% (25.2-36.2) | 87.6% |
| Addis Ababa | 28.0% (14.0-44.7) | 96.6% |
| National level | 28.0% (20.5-36.1) | 97.1% |
| Tigray | 26.5% (23.1-30.0) | - |
| Harar | 22.9% (20.3-25.5) | - |
| Overall | 26.5%(24.8-28.2) | 88.8% |
| **By data collection period** | | |
| 2014 and before | 28.2% (25.1-31.4) | 94.6% |
| After 2014 | 28.8% (23.7-34.0) | 94.5% |
| Overall | 28.4% (25.7-31.1) | 87.2% |

We assessed the issue of publication bias by visual inspection of funnel plot and by using the Egger’s regression test. Though the funnel plot looks asymmetrical (**Fig 3**), the Egger's test

showed that no relationship between the effect size and its precision (P-value = 0.776). This might be due to heterogeneity in true effects.

**Fig 3**: Funnel plot of the prevalence of unintended pregnancy in Ethiopia.

### Determinants of unintended pregnancy in Ethiopia

The significant determinants of unintended pregnancy reported from each study are presented in (**Table S2**).

In six studies, researchers examined the association between spousal communication about family planning and unintended pregnancy. Of this, five studies (26,30,36,37,41) showed that spousal communication about family planning was associated with an unintended pregnancy. The result of this meta-analysis showed that not communicating with one's husband about family planning was more likely to lead to unintended pregnancy (OR: 3.56, 95%CI: 1.68-7.53) (**Fig 4**).

Ten studies were examined the association between the use of modern family planning methods and unintended pregnancy(10,22,26,29,34,37–39,41,42). Consequently, the result of this study showed that unintended pregnancy is more likely among women who never used family planning methods (OR: 2.08, 95%CI: 1.18-3.69) (**Fig.5**).

Of 21 studies that examined the association of maternal education level with unintended pregnancy, ten studies reported that maternal education status was associated with an unintended pregnancy (29,31,32,34–36,38–40). Furthermore, among the included studies, women's age, time to reach the nearest health facility, household wealth, and marital status showed significant association with unintended pregnancy.

**Fig 4**. The pooled odds ratio of the association between spousal communication about family planning and unintended pregnancy in Ethiopia.

**Fig 5**. The pooled odds ratio of the association between the use of family planning and unintended pregnancy in Ethiopia.

## Discussion

Unintended pregnancy has significant consequences for the health and welfare of women and children (7). Pregnancy intention in developing countries is influenced by socio-cultural, environmental, individual and health service-related factors (43). This systematic review and meta-analysis was conducted to determine the overall prevalence of unintended pregnancy and to identify its determinants in Ethiopia**.**

We found that the overall prevalence of unintended pregnancy in Ethiopia was 28% (95% CI: 25-31). This finding is consistent with the 2016 EDHS result (4), which reported that from all births in the past 5 years and current pregnancies, one-fourth (25%) were unintended. Additionally, in the previous two consecutive EDHS reports, we noted that the proportion of women who want no more births declined from 42% in 2005 to 37% in 2011. The prevalence of unintended pregnancy found in this study agreed with other previous studies conducted in Egypt (44), Ghana (45), and Bangladesh (9), which reported that nearly one-third (30.7%), 29.8% and 29% of pregnancies were unintended, respectively. However, the magnitude of unintended pregnancy found in this study was lower than previous studies conducted in Malawi, South Africa, and the Republic of Congo (46–49). This variation might be attributed to methodological differences in the assessment of pregnancy intention. The other possible reason for inconsistency in the prevalence of unintended pregnancy could be explained by the difference in the socio-economic characteristics of study participants (50–52).

In this study, we found that the pooled prevalence of unintended pregnancy was 26.4% and 30% for community-based cross-sectional and institutional-based cross-sectional studies, respectively. This variation could be explained by institution-based studies that include women who visit health facilities for legal abortion services, which is probably unintended pregnancy. About 97 % of women seeking an abortion reported having an unintended pregnancy (50). The highest prevalence of unintended pregnancy was observed from the Oromiya region (33.8%), and the lowest was in Harar (22.9%). The possible explanation might be due to the difference in the utilization of family planning methods.

In this systematic review and meta-analysis, we noted that a number of factors were found to be associated with an unintended pregnancy. Consequently, we found that women who hadn't communicated with their husbands about family planning methods were more likely to have an unintended pregnancy. The possible explanation for this result might be women's perception that their husbands oppose family planning, which is one of the dominant factors for discouraging the contraceptive practice in a wide variety of settings (20). Partner awareness of contraceptive and open discussion about family planning methods will decrease the risk of unintended pregnancy. The result of this study also showed that unintended pregnancy is more likely among women who never used family planning methods. This is due to the fact that awareness and proper utilization of modern contraceptives are crucial to reduce unintended pregnancy.

In this systematic review, unintended pregnancy was less likely among married women. The possible reason for this result might be that, in Ethiopian culture, pregnancy without marriage is unacceptable in most communities. This result is supported by previous studies conducted in South Africa and Kenya (53,54).

Though previous evidence stated that youngest women experience the highest rate of unintended pregnancy (55), in this systematic review, we observed controversial results regarding the association between women's age and unintended pregnancy. Some of the studies reported that younger women are more likely to have an unintended pregnancy, while other studies showed that older women are more likely to have unintended pregnancy.

Due to the inconsistent classification of maternal education level, time-lapse to reach the nearest health facility, parity, occupation and religion, we couldn't pool quantitatively, in this systematic review and meta-analysis. Contradictory results are observed between individual studies. Four studies (29,32,36,40) reported that women who have relatively better education were less likely to have an unintended pregnancy as compared to those who didn't have formal education. It is reasonable that as educational level increases the awareness of reproductive health also increases. Additionally, as the educational level increases, the spousal communication on pregnancy, awareness of long-term family planning, and receiving adequate ANC also increases. However, two studies reported that women who had relatively better education were more likely to have unintended pregnancy (31,39).

Moreover, in this study, we observed that as the time-lapse to reach the nearest health facility for providing contraceptives increased, women were more likely to have unintended pregnancy (10,29,42). It is also reasonable that as the time-lapse to reach the nearest health facility increases, the probability of missing ANC visits also increase, which in turn is associated with unintended pregnancy (13,20).

In this study, we noted that the parity of respondents showed a significant relationship with an unintended pregnancy. Consequently, multiparous women were more likely to experience an unintended pregnancy than nulliparous (34,35,40,56). This might be because ~~of~~ women who have attained their desired number of children will perceive any additional child as unwanted (57). Furthermore, the pregnancy of unemployed women is more likely to be unintended than employed women's (29,37).

Moreover, in this study, another important variable which significantly associated with unintended pregnancy was religion. Muslim mothers were less likely to report having an unintended pregnancy as compared to orthodox mothers. This finding is consistent with a study conducted in Ghana (58). This might be as a result of doctrinal differences among the women of along with different religions.

## Limitations

This study was not without limitations. Firstly, the review was limited to only articles published in the English language. Secondly, all of the included studies were cross-sectional, which limits assessment of the cause-effect relationships. Thirdly, we were unable to show the pooled odds ratio for all variables associated with unintended pregnancy because the included studies classified the variables in different ways.

## Conclusions

In this study, a high prevalence of unintended pregnancy was observed. Lack of spousal communication, never using family planning, maternal education, and household wealth level were significantly associated with an unintended pregnancy. This study implies the need to develop plans and policies to improve the awareness of contraceptive utilization and strengthen spousal communication related to pregnancy. Emphasis should be given to those women at a distant from health facilities, unmarried and teenagers.

## Refernces


1. Santelli BJ, Rochat R, Hatfield- K, Gilbert C, Curtis K, Cabral R, et al. The Measurement and Meaning of Unintended Pregnancy. 1999;94–101.
2. Unintended Pregnancy Prevention | Unintended Pregnancy - CDC-(Dec 23, 2016 - https://www.cdc.gov/reproductivehealth/contraception/unintendedpregnancy/index.htm.
3. Bearak J, Popinchalk A, Alkema L, Sedgh G. Global , regional , and subregional trends in unintended pregnancy and its outcomes from 1990 to 2014 : estimates from a Bayesian hierarchical model. Lancet Glob Heal [Internet]. 2014;6(4):e380–9. Available from: http://dx.doi.org/10.1016/S2214-109X(18)30029-9
4. Central Statistical Agency (CSA) [Ethiopia] and ICF. 2016. Ethiopia Demographic and Health Survey 2016. Addis Ababa, Ethiopia, and Rockville, Maryland, USA: CSA and

ICF.

5. Singh A, Singh A, Thapa S. Adverse Consequences of Unintended Pregnancy for Maternal and Child Health in Nepal. 2015;
6. Kabir, Sandra M.: Causes and Consequences of Unwanted Pregnancy from Asian Women's Perspectives. International Journal of Obstetrics. 1989. Supple 3 .p.9-14.
7. Mumah, J, Kabiru, CW, Mukiira, C, Brinton, J, Mutua, M, Izugbara, C, Birungi, H. and Askew, I. 2014. "Unintended Pregnancies in Kenya: A Country Profile," STEP UP Research Report. Nairobi: African Population and Health Research Center.
8. Induced Abortion and Postabortion Care in Ethiopia,Ipas,January 2017.
9. Rahman M, Rahman M, Tareque I, Ferdos J. Maternal Pregnancy Intention and Professional Antenatal Care Utilization in Bangladesh : A Nationwide Population-Based Survey. 2016;1–15.
10. Melese KG, Gebrie MH, Badi MB, Mersha WF. Unintended Pregnancy in Ethiopia : Community Based Cross-Sectional Study. 2016;2016.
11. Basinga P, Moore AM, Singh S, Remez L, Birungi F, Nyirazinyoye L. Unintended Pregnancy And Induced Abortion In Rwanda:Causes and Consequences. Guttmacher Inst.
12. Wado YD, Afework MF, Hindin MJ. Effects of Maternal Pregnancy Intention , Depressive Symptoms and Social Support on Risk of Low Birth Weight : A Prospective Study from Southwestern Ethiopia. 2014;9(5):1–7.
13. Wado YD, Afework MF, Hindin MJ. Unintended pregnancies and the use of maternal health services in southwestern Ethiopia. BMC Int Health Hum Rights [Internet]. 2013;13(1):1. Available from: BMC International Health and Human Rights
14. Cochrane Book Series. Cochrane Handbook for Systematic Reviews of Interventions.
15. Hoy D, Brooks P, Woolf A, Blyth F, March L, Bain C, et al. Assessing risk of bias in prevalence studies : modification of an existing tool and evidence of interrater agreement. J Clin Epidemiol. 2012;65(9):934–9.
16. Wang N. How to Conduct a Meta-Analysis of Proportions in R : A Comprehensive Tutorial Conducting Meta-Analyses of Proportions in R. 2018;(June).
17. Higgins JPT, Thompson SG, Deeks JJ, Altman DG. Measuring inconsistency in meta-analyses. :557–60.
18. Abame DE, Abera M, Tesfay A, Yohannes Y, Ermias D, Markos T, et al. Relationship

Between Unintended Pregnancy and Antenatal Care Use During Pregnancy in Hadiya Zone, Southern Ethiopia. J Reprod Infertil. 2019;20(1):42–51.

19. Worku S, Fantahun M. Unintended pregnancy and induced abortion in a town with accessible family planning services : The case of Harar in eastern Ethiopia.

20. Sectional C, Abayu H, Birhanu Z, Nega A, Kidanemariam A. Prevalence and Associated Factors of Unintended Pregnancy in Welkaite. 2015;2(2).

21. Admasu E, Mekonnen A, Setegn T, Abeje G. Level of unintended pregnancy among reproductive age women in Bahir Dar city administration , Northwest Ethiopia. BMC Res Notes. 2018;1–5.

22. Fite RO, Mohammedamin A, Abebe TW. Unintended pregnancy and associated factors among pregnant women in Arsi Negele. BMC Res Notes. 2018;1–7.

23. Gite A, Liulseged N, Seyife H, Abrha Y, Workineh Y, Shegaze M, et al. Unintended Pregnancy: Magnitude and Associated Factors among Pregnant Women in Arba Minch Town, Gamo Gofa Zone, Ethiopia, 2015. Reprod Syst Sex Disord Curr Res. 2016;5(4):6.

24. Hamdela B, Abebe G, Tilahun T. Unwanted Pregnancy and Associated Factors among Pregnant Married Women in Hosanna Town , Southern. 2012;7(6).

25. Teshome FT, Hailu AG, Teklehaymanot AN. Prevalence of unintended pregnancy and associated factors among married pregnant women in Ganji woreda , west Wollega Oromia region , Ethiopia. 2014;2(2):92–101.

26. Tsegaye AT, Mengistu M, Shimeka A. Prevalence of unintended pregnancy and associated factors among married women in west Belessa Woreda , Northwest. 2018;1–8.

27. Yenealem F, Niberet G. Prevalence and associated factors of unintended pregnancy among pregnant woman in Gondar town , North west Ethiopia ,. BMC Res Notes. 2019;1–5.

28. Health H, Shewa N. Assessment of the Prevalence of Unplanned Pregnancy and Associated Factors among Pregnant Women Attending Antenatal Care Unit at Journal of Women ’ s Health Care. 2017;6(6).

29. Darega B, Dida N. Unplanned Pregnancy: Prevalence and Associated Factors among Antenatal Care Attending Women in Bale Zone, Oromiya Region, Southeast Ethiopia: A Facility - based Cross Sectional Study. 2015;15(4).

30. Liyew B, Desalegn B, Ayalew Y. Prevalence of Unintended Pregnancy and Associated

Factors among Currently Pregnant Mothers in Kombolcha, Southern Wollo, Ethiopia, 2017.
31. Feyisso M, Girma A, Yimam H, Hailu S. Journal of Women ' s Health Care Magnitude of Unintended Pregnancy and its Determinants among Pregnant Women Visiting Dilla University Referral Hospital , South. 2017;6(4).
32. Gebreamlak W, Aragaw A, Lemma S, Demilew W. Magnitude and factors influencing unintended pregnancy among pregnant women attending antenatal care at Felege Hiwot referral hospital , Northwest Ethiopia : A cross-sectional study. 2014;2(4):261–9.
33. Gizaw W, Fetene G, Mohammed S, Alemu A. iMedPub Journals Magnitude of Unplanned Pregnancy and its Associated Factors among Pregnant Women Attending Antenatal Care at Tepi General Hospital Sheka. 2018;1–8.
34. Goshu YA, Yitayew AE (2019) Prevalence and determinant factors of unintended pregnancy among pregnant women attending antenatal clinics of Addis Zemen hospital. PLoS ONE 14(1): e0210206. https://doi.org/10.1371/journal. pone.0210206.
35. Kahasay S, Shemelis T. Assesment of magnitude and factors associated with unintended pregnancy among preparatory highschool students,Addis Ababa,Ethiopia. 2015
36. Kassie T, Geteneh M, Ali A, Tefera W. Magnitude and factors associated with unintended pregnancy among pregnant women in Addis Ababa, Ethiopia. Glob J Med PUBLIC Heal. 2017;6(4):15.
37. Kibret A, Bayu H, Merga M. Prevalence of Unintended Pregnancy and Associated Factors among Pregnant Women Attending Antenatal Clinics in Debre-markos Town, North West Ethiopia 2012. Women's Heal Care. 2014;4(3).
38. Mulat S, Fekadu M, Abera H, Bekele G, Bedaso A. Journal of Women ' s Health Care Prevalence of Unplanned Pregnancy and Associated Factors among Mothers Attending Antenatal Care at Hawassa City Public Hospitals , Hawassa , SNNPR ,. 2017;6(4).
39. Habte D, Teklu S, Melese T, Magafu MGMD. Correlates of Unintended Pregnancy in Ethiopia : Results From a National Survey. 2013;8(12):1–8.
40. Tebekaw Y, Aemro B, Teller C. Prevalence and determinants of unintended childbirth in Ethiopia. 2014;1–9.
41. Abayu H, Birhanu Z, Nega A, Kidanemariam3 A. Prevalence and Associated Factors of Unintended Pregnancy in Welkaite Woreda, Tigray and North Ethiopia Cross Sectional

Study by 2012. J Pregnancy Child Heal. 2015;2(2).

42. Kassa N, Berhane Y, Worku A. Predictors of unintended pregnancy in Kersa ,. 2012;1–7.

43. Yakubu I, Salisu WJ. Determinants of adolescent pregnancy in sub-Saharan Africa: a systematic review. BMC Reprod Heal. 2018;

44. Mohamed EAB, Hamed AF, Yousef FMA, Ahmed EA. Prevalence , determinants , and outcomes of unintended pregnancy in Sohag district , Egypt. 2019;9.

45. Ameyaw EK. Prevalence and correlates of unintended pregnancy in Ghana: Analysis of 2014 Ghana Demographic and Health Survey. Matern Heal Neonatol Perinatol. 2018;4(1):1–6.

46. Palamuleni ME, Adebowale AS. Prevalence and determinants of unintended pregnancies in Malawi. African Population Studies. 2014 Apr 29;28(1):551-63.

47. Adeniyi OV, Ajayi AI, Moyaki MG, Goon D Ter, Avramovic G, Lambert J. High rate of unplanned pregnancy in the context of integrated family planning and HIV care services in South Africa. BMC Health Serv Res. 2018;18(1):4–11.

48. Hall JA, Barrett G, Phiri T, Copas A, Malata A, Stephenson J (2016) Prevalence and Determinants of Unintended Pregnancy in Mchinji District, Malawi; Using a Conceptual Hierarchy to Inform Analysis. PLoS ONE 11(10): e0165621. doi:10.1371/journal.pone.01656.

49. Ndziessi G, Bitemo M, Kaboru BB. Unintended Pregnancies and Associated Factors among Contraceptive Users: a Study from Referral Hospitals in Brazzaville, Republic of Congo. SM J Community Med. 2016;2(1):1015.

50. Metcalfe A, Talavlikar R, Du Prey B, Tough SC. Exploring the relationship between socioeconomic factors, method of contraception and unintended pregnancy. Reprod Health [Internet]. 2016;13(1):1–8. Available from: http://dx.doi.org/10.1186/s12978-016-0151-y

51. Omani-Samani R, Amini Rarani M, Sepidarkish M, Khedmati Morasae E, Maroufizadeh S, Almasi-Hashiani A. Socioeconomic inequality of unintended pregnancy in the Iranian population: A decomposition approach. BMC Public Health. 2018;18(1):1–8.

52. Roy TK, Singh BP (2016). Correlates of Unwanted Births in Bangladesh: A Study through Path Analysis. PLoS ONE 11(10): e0164007. https://doi.org/10.1371/journal.pone.0164007.

53. Africa S, Haffejee F, Connor LO, Govender N, Reddy P, Sibiya MN, et al. Factors associated with unintended pregnancy among women attending a public health facility in Factors associated with unintended pregnancy among women attending a public health facility in KwaZulu-Natal , South Africa. South African Fam Pract [Internet]. 2018;6190:1–5. Available from: http://doi.org/10.1080/20786190.2017.1396790
54. Ikamari L, Izugbara C, Ochako R. Prevalence and determinants of unintended pregnancy among women in Nairobi , Kenya. 2013;1–9.
55. Finer LB et al. Contraception. 2011;84:478–485.
56. Mohammed F, Musa A, Amano A. Prevalence and determinants of unintended pregnancy among pregnant woman attending ANC at Gelemso General Hospital , Oromiya Region , East Ethiopia : a facility based cross-sectional study. BMC Womens Health [Internet]. 2016;10–6. Available from: http://dx.doi.org/10.1186/s12905-016-0335-1
57. Addis K, Bayu H, Merga M. Prevalence of Unintended Pregnancy and Associated Factors among Pregnant Women Attending Antenatal Clinics in Debre-markos Town, North West Ethiopia 2012. Women's Heal Care. 2014;4(3):6.
58. Ameyaw EK. Prevalence and correlates of unintended pregnancy in Ghana: Analysis of 2014 Ghana Demographic and Health Survey. Maternal health, neonatology and perinatology. 2018 Dec;4(1):17.

**Table S1**: Assessing the risk of bias for the included studies

**Table S2**: Significant determinants of unintended pregnancy reported from each study.

 Click here to access/download;Figure;Fig 1.docx

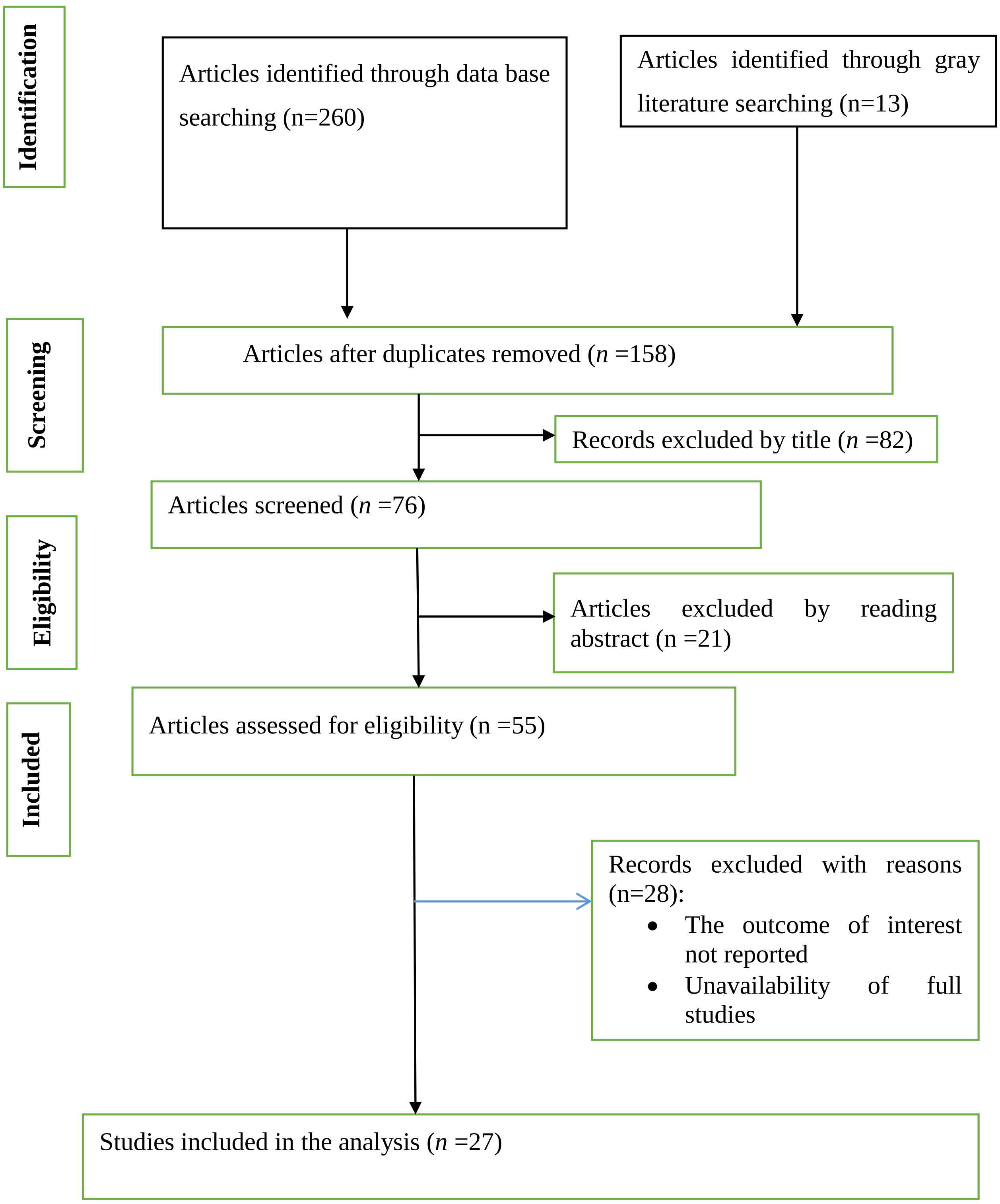


**Figure 1**: Flow chart diagram describing the selection of studies included in the systematic review and meta-analysis of prevalence and determinants of unintended pregnancy in Ethiopia.

Figure Click here to access/download;Figure;Fig 2.docx

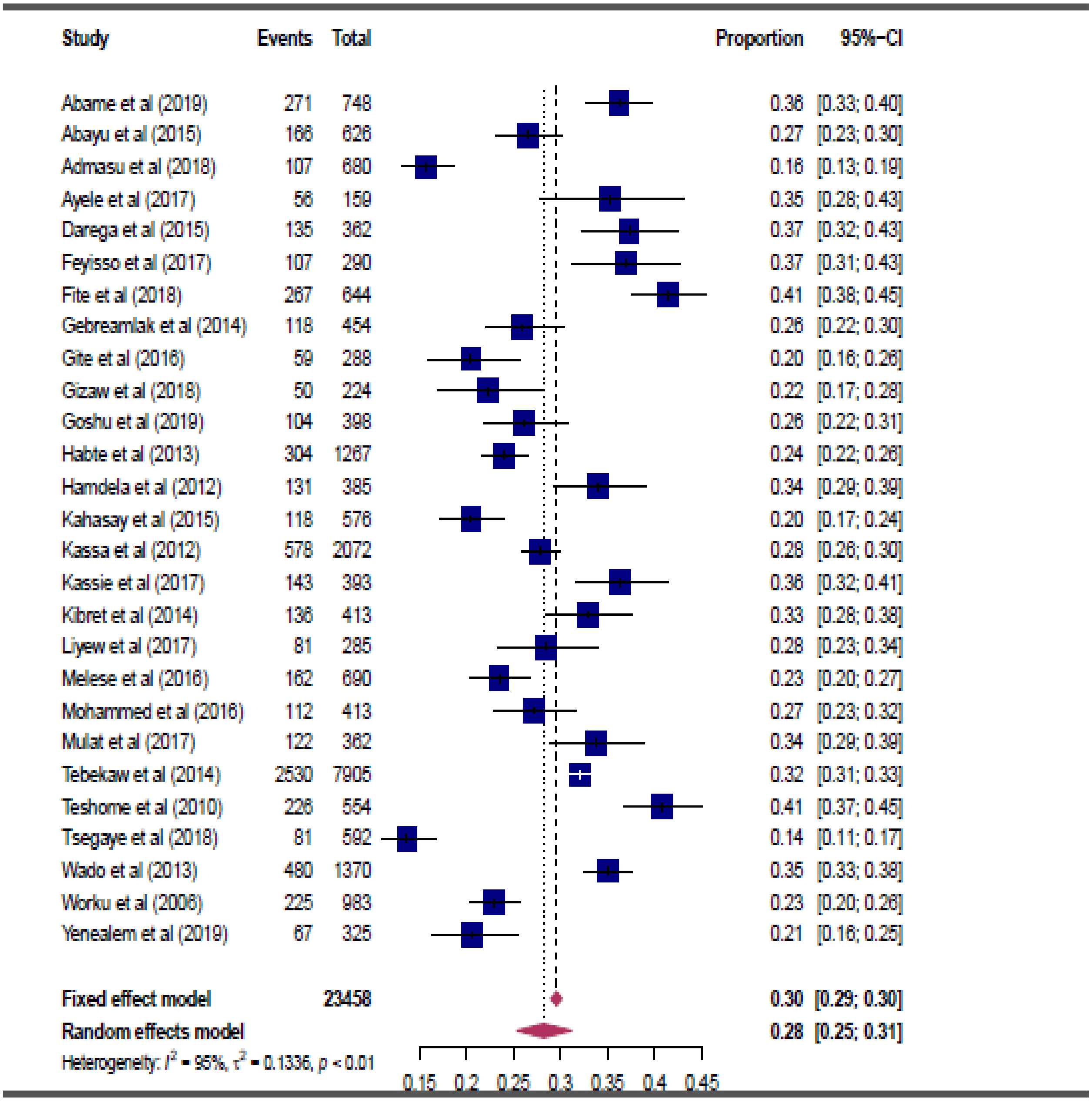


**Fig 2**. Forest plot of the pooled prevalence of unintended pregnancy in Ethiopia

 Click here to access/download;Figure;Fig 3.docx

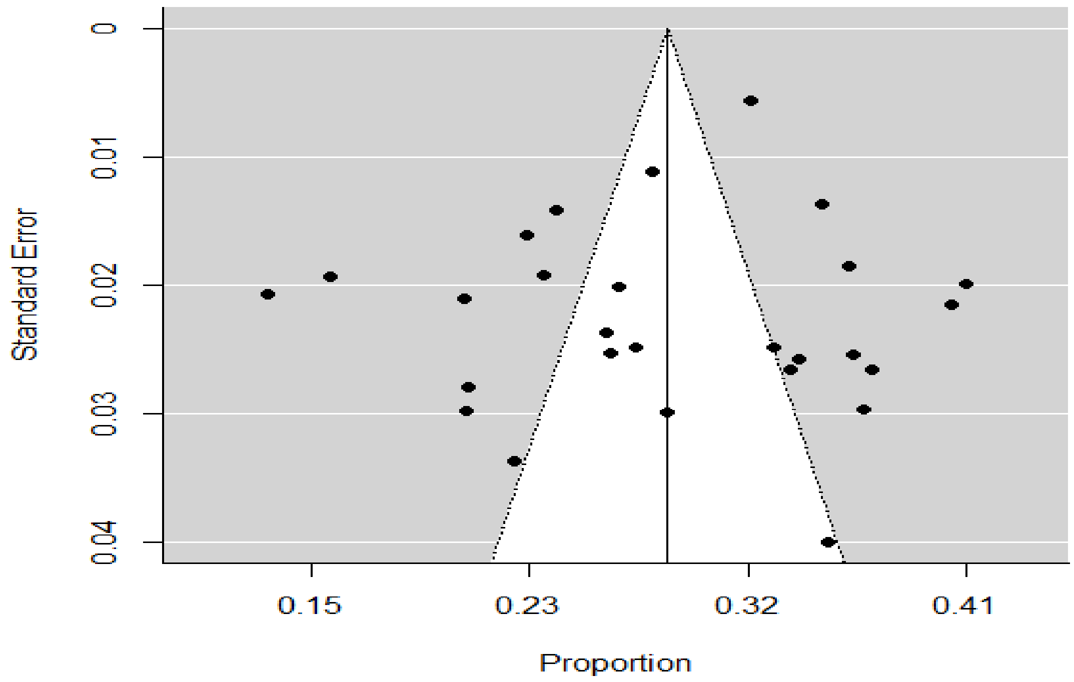


**Fig 3**: Funnel plot of the prevalence of unintended pregnancy in Ethiopia

Figure
Click here to access/download;Figure;Fig 4.docx

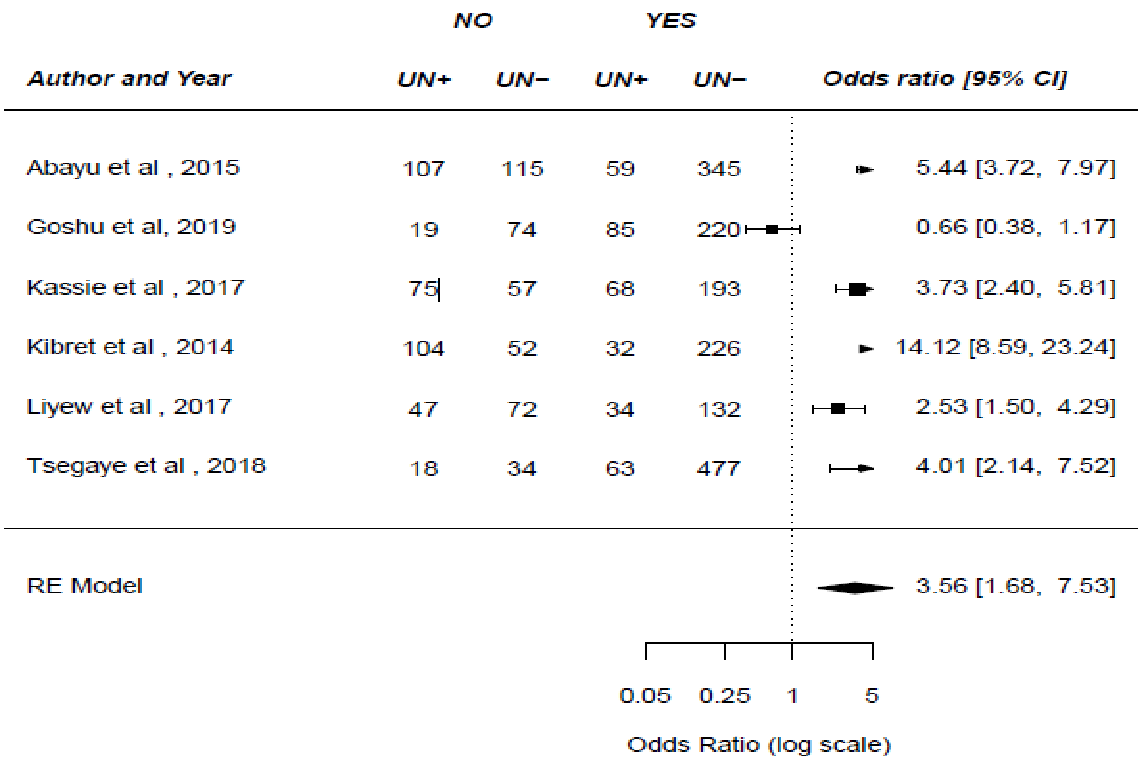


**Fig 4**. The pooled odds ratio of the association between spousal communication about family planning and unintended pregnancy in Ethiopia.

Figure Click here to access/download;Figure;Fig 5.docx

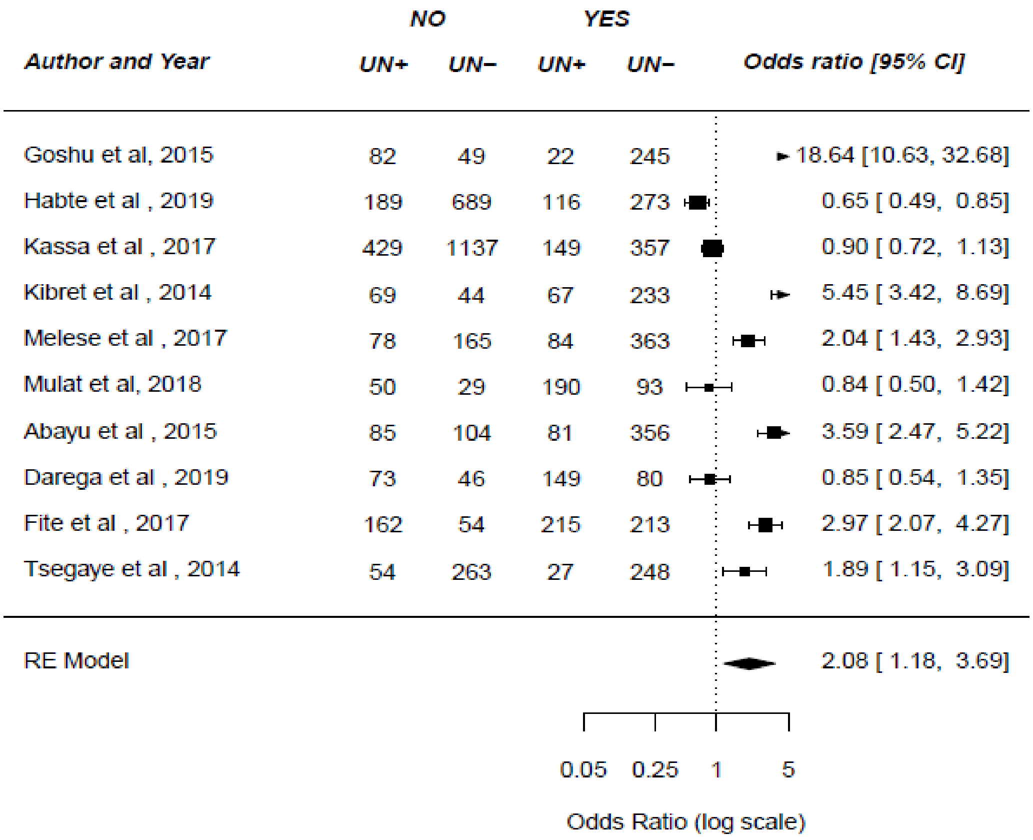


**Fig 5**. The pooled odds ratio of the association between the use of family planning and unintended pregnancy in Ethiopia.

Click here to access/download
**Supporting Information**
Prisma checklist.doc

# Prevalence and determinants of unintended pregnancy in Ethiopia: a systematic review and meta-analysis of observational studies

Muluneh Alene[1*], Leltework Yismaw[1], Yebelay Berelie[2], Bekalu Kassie[3], Reta Yeshambel[4], Moges Agazhe Assemie[1]

1Department of Public Health, Debre Markos University, Debre Markos, Ethiopia
2Department of Statistics, Debre Markos University, Debre Markos, Ethiopia
3Department of Midwifery, Debre Markos University, Debre Markos, Ethiopia
4Department of Biology, Mizan-Tepi University, Teppi, Ethiopia

**Author's email addresses**
MA: mulunehadis@gmail.com
LY: lielt.yismaw@gmail.com
YB: yebelay.ma@gmail.com
BK: bekalukassiedmu@gmail.com
RY: reta.yeshambel@gmail.com
MAA: agazhemoges@gmail.com

**[*]Corresponding author**
mulunehadis@gmail.com

**Abstract**

**Background:** Unintended pregnancy has significant consequences for the health and welfare of women and children. Despite this, a number of studies with inconsistent findings were conducted to reduce unintended pregnancy in Ethiopia; unavailability of a nationwide study that determines the prevalence of unintended pregnancy and its determinants is an important research gap. Thus, this study was conducted to determine the overall prevalence of unintended pregnancy and its determinants in Ethiopia.

**Methods:** We searched from Google Scholar, PubMed, Science Direct, Web of Science, CINAHL, and Cochrane Library databases for studies. Each of the original studies was assessed using a tool for the risk of bias of observational studies. The heterogeneity of studies was also assessed using $I^2$ test statistics. Data were pooled and a random effect meta-analysis model was fitted to provide the overall prevalence of unintended pregnancy and its determinants in Ethiopia. In addition, the subgroup analyses were performed to investigate how the prevalence of unintended pregnancy varies across different groups of studies.

**Results:** Twenty-seven studies that satisfy the eligibility criteria were included. We found that the overall prevalence of unintended pregnancy in Ethiopia was 28% (95% CI: 25-31). The subgroup analysis showed that the highest prevalence of unintended pregnancy was observed from the Oromiya region (33.8%) followed by Southern Nations, Nationalities, and Peoples' region (30.6%), and the lowest was in Harar. In addition, the pooled prevalence of unintended pregnancy was 26.4% (20.8-32.4) and 30.0% (26.6-33.6) for community-based cross-sectional and institution-based cross-sectional studies respectively. The pooled analysis showed that not communicating with one's husband about family planning was more likely to lead to unintended pregnancy (OR: 3.56, 95%CI: 1.68-7.53). The pooled odds ratio also showed that unintended pregnancy is more likely among women who never use family planning methods (OR: 2.08, 95%CI: 1.18-3.69). Furthermore, the narrative review of this study showed that maternal education, age, and household wealth index are strongly associated with an unintended pregnancy.

**Conclusions:** In this study, the prevalence of unintended pregnancy was high. Lack of spousal communication, never using family planning, maternal education, and household wealth level

were significantly associated with an unintended pregnancy. This study implies the need to develop plans and policies to improve the awareness of contraceptive utilization and strengthen spousal communication related to pregnancy.

**Key words:** Prevalence, Unplanned pregnancy, Ethiopia, Meta-analysis, Maternal morbidity and mortality

## Introduction

Unintended pregnancy is a pregnancy which is either mistimed or unwanted (1,2). It is a public health problem and a risk factor for adverse health outcomes, particularly for maternal and child health (3). Though the rate of unintended pregnancy fell worldwide between 1990 and 2014, it dropped less sharply in developing regions than in developed regions (3). Ethiopia is one of the developing countries with a high prevalence of unintended pregnancy. In Ethiopia, more-than one-third (38%) of pregnancies were unintended in 2014; slightly lower than in 2008 which was 42%. According to results from the 2016 Ethiopian Demographic and Health Survey (EDHS) of all births in the past five years and current pregnancies, 25% are unintended. Also, the 2016 EDHS report showed that the overall difference between the wanted fertility rate and the total fertility rate is one child, which suggests that Ethiopian women are currently having, on average, one child more than they want (4).

Pregnancies should be planned before conception unless a woman may not be in optimal health for childbearing (2). Unintended pregnancy leads to maternal mortality and morbidity due to the complications of unsafe abortion, miscarriage, and unplanned births, which burdens the health system at all (5–7). Annually, more than 1 in 10 pregnancies end in abortion, and 1 in 27 mothers die due to the complications of pregnancy or childbirth in Ethiopia (8). A woman with an unintended pregnancy will have low physical and mental health, low self-care, and depression during pregnancy. These lead to poor Antenatal Care (ANC) service utilization and postpartum depression, which is a risk factor for unfavorable pregnancy outcome, and maternal morbidity and mortality (7,9). Consequently, the newborns of unintended pregnancies are faced with low birth weight and inadequate vaccinations which increases the risk for childhood illnesses (5,7,10–12). Previous evidence showed that unintended pregnancy mainly results from inconsistent or incorrect use of contraceptive methods, and women's are less likely than men to want more children no matter how many children they already have (2,13).

In Ethiopia, a number of studies were conducted to estimate the magnitude and to identify the determinants of unintended pregnancy. However, the reported prevalence and determinants in these fragmented studies vary depending on the characteristics of study participants, the type of design employed and the variables analyzed. Combined findings of existing studies significantly strengthen the quality of evidence investigating the national prevalence and determinants of unintended pregnancy. Thus, this systematic review and meta-analysis was conducted to determine the overall prevalence and determinants of unintended pregnancy in Ethiopia. This review is intended to bring an improvement in the design of future studies related to unintended pregnancy. The findings of this study are also intended to improve health worker's intervention in the area of reproductive health.

## Materials and methods

### Study design and setting

A systematic review and meta-analysis, which aimed to estimate the overall prevalence of unintended pregnancy and its determinants was conducted in Ethiopia. Ethiopia is situated in the horn of Africa, and bordered by Eritrea to the north, Sudan and South Sudan to the west, Kenya to the south, and Djibouti and Somalia to the east. Nearly eight in ten women (78%) live in rural areas in Ethiopia, and half of women age 15-49 (48%) have no education (4).

### Eligibility criteria

In this systematic review and meta-analysis, studies were included with the following criteria: 1) only studies conducted in Ethiopia. 2) Only studies reported in English language, 3) only studies involving pregnant women or had given birth at least once preceding the survey and 4) all observational studies reporting the prevalence and determinants of unintended pregnancy. Both published and unpublished articles were included. Studies which were not fully accessible were excluded because of the inability to assess the quality of studies without their full text.

### Searching for studies

A comprehensive search strategy was done by three (MA, LY, and RY) of the authors. Both published and unpublished articles on unintended pregnancy were searched from international (Google Scholar, PubMed, Science Direct, Web of Science, CINAHL, and Cochrane Library), and national (Ethiopian Journal of Public Health and Nutrition) electronic databases. First,

articles were searched by examining the full titles ("Prevalence and determinants of unintended pregnancy in Ethiopia") and then keywords (unintended pregnancy, unplanned pregnancy, unwanted pregnancy, mistimed pregnancy, determinants, risk factors, associated factors, Ethiopia). These keywords were used separately and in combination using Boolean operators "OR" or "AND". In addition, we searched from the reference lists of all the included studies (snowball technique) to identify any other studies that may have been missed by our search strategy. Finally, all studies were imported into reference management software (Mendeley Desktop).

**Outcome measures and data extraction**

Unintended pregnancy, which is either mistimed or unwanted, was the primary outcome of the study. Unwanted pregnancy occurred when a woman did not want to have any more pregnancies, whereas mistimed pregnancy is a pregnancy that was wanted by the woman at some time, but which occurred sooner than they wanted. The pregnancy intention for the included studies was assessed by interviewing current pregnant women or women who had given birth at least once preceding the survey.

All essential data from the included studies were extracted independently by two (MA and LY) of the authors using a predesigned data abstraction form. This form includes the last name of the first author, publication year, data collection period, study design, region of the study conducted, study population, sample size, response rate, and the magnitude of unintended pregnancy. In addition, we extracted the adjusted odds ratios with corresponding 95% confidence intervals to measure the strength of effects. Deviation in a data extraction process was resolved by discussion and consensus involving all authors.

**Quality assessment for studies**

The quality of meta-analysis depends on the included studies (14). Two authors (MA and YB) assessed the risk of bias for the included studies using the tool of risk of bias assessment for observational studies (15). This tool includes 10 items. The first four items assess the external validity, while the other six items evaluate the internal validity of the study. All items of the tool were filled in for each included study and categorized as low risk bias (if the response is "yes"), higher risk (if the response is "no"), and not clear. The quality of the study was determined by summing the score given for each item. Lastly, unclear risk of bias was categorized as high risk

of bias then; the summary assessment risk of bias for each study was categorized according to the number of high risk of bias: low (≥2), moderate (3-4), and high (≥5).

**Data processing and analysis**

After extracting all relevant data using Microsoft excel software, data were exported to R statistical software for meta-analysis. The double arcsine transformation which stabilizes the sampling variance was applied to estimate the weighted average prevalence, and the transformed summary prevalence are converted back for ease of interpretation (16).

We assessed the consistency of studies using $I^2$ test statistics (17). This test examines the null hypothesis that all the included studies are evaluating the same effect. Consequently, since there was heterogeneity between the original studies ($I^2$=96%, p<0.01) a random effect model is needed, and to account for between-study variance a random effect meta-analysis with an estimation of DerSimonian and Laird method was performed. The possible sources of heterogeneity among studies might be differences in study participants, study design, risk of bias and data collection period. Furthermore, subgroup analyses were conducted to investigate how the prevalence of unintended pregnancy varied across different subgroups of studies.

## Results

**Search results**

Our comprehensive search for studies was between the 15th of February and the 30th of April, 2019. The flow chart diagram that shows our literature search, study selection, and the number of included studies is presented in (**Fig 1**). Initially, a total of 273 articles were identified during our search, and then 115 articles were excluded due to duplication. Finally, 27 studies that satisfied the eligibility criteria were included in this systematic review and meta-analysis.

**Figure 1**: Flow chart diagram describing the selection of studies included in the systematic review and meta-analysis of prevalence and determinants of unintended pregnancy in Ethiopia.

**Description of the included studies**

The detail descriptions of the included studies are shown in (**Table 1**). All studies included in this review were conducted between 2006 and 2019. Of all the included studies, eleven (10,18–27) were community-based cross-sectional, while twelve were institutional-based cross-sectional studies (28–38). The data for the two studies were also taken from the 2011 EDHS (39,40). The

majority of respondents of the original studies were pregnant mothers. The number of participants in each study varied from the lowest of 165 (28) to the highest of 7,759 (40). A study with the smallest study participants was an institutional-based cross-sectional study, while the data for the highest study participants were taken from the 2011 EDHS. Furthermore, nine studies were conducted in the Amhara region, six studies in the Oromiya region, six studies in Southern Nations, Nationalities, and Peoples' Region (SNNPR), two studies in Addis Ababa, one study in Harar, and two studies from the national survey. Moreover, the prevalence of unintended pregnancy ranged from 13.7% (26) to 41.5% (22).

**Table 1**: Descriptions of the included studies conducted in Ethiopia on unintended pregnancy.

| **First author (publication year)** | **Study area (Region)** | **Study design** | **Data collection period** | **Study population** | **Sample size** | **Response rate** | **Prevalence of unintended pregnancy** |
|---|---|---|---|---|---|---|---|
| Abame et al (2019) | SNNPR | CBCS | March 13, 2017 to April 13, 2017 | pregnant mothers | 748 | 97% | 36.2% |
| Abayu et al (2015) | Tigray | CBCS | 24/09/2012 to 18/10/2012 | Pregnant women | 626 | 96.3% | 26% |
| Admasu et al (2018) | Amhara | CBCS | NR | pregnant women or with under 1 child age | 680 | 91% | 15.8% |
| Ayele et al (2017) | Oromiya | IBCS | June 10, 2017 to July 24, 2017 | pregnant women following ANC | 165 | 96% | 35.2% |
| Darega et al (2015) | Oromiya | IBCS | May ,2014 | Women following ANC | 362 | 100% | 37.3% |
| Feyisso et al (2017) | SNNPR | IBCS | February to June, 2016 | Women following | 290 | NR | 36.9% |

| | | | | | | | |
|---|---|---|---|---|---|---|---|
| | | | | ante natal and post natal ANC | | | |
| Fite et al (2018) | Oromiya | CBCS | May 01–July 30, 2017 | Pregnant women | 704 | 91.5% | 41.5% |
| Gebreamlak et al (2014) | Amhara | IBCS | June to July 2012 | Pregnant women | 454 | NR | 26% |
| Gite et al (2016) | SNNPR | CBCS | February 15-March 11,2015 | Pregnant women | 311 | 95.4% | 19.4% |
| Gizaw et al (2018) | SNNPR | IBCS | February 24 to April 24, 2017 | Pregnant women | 224 | 100% | 22.3% |
| Goshu et al (2019) | Amhara | IBCS | April 01 to May 30, 2018 | Pregnant women | 398 | 100% | 26.1% |
| Habte et al (2013) | National level | EDHS | NA | Pregnant women | 1267 | NA | 24% |
| Hamdela et al (2012) | SNNPR | CBCS | April 02 to 15, 2011 | pregnant married women | 385 | 100% | 34% |
| Kahasay et al (2015) | Addis Abeba | IBCS | NR | female students aged from 16-19 years | 576 | 100% | 20.4% |
| Kassa et al | Oromiya | KDSH | December | Pregnant | 2072 | 100% | 27.9% |

| | | | | | | | |
|---|---|---|---|---|---|---|---|
| (2012) | | RC | 2009 to November 2010 | women | | | |
| Kassie et al (2017) | Addis Abeba | IBCS | February to May 2015 | Pregnant women | 393 | 100% | 36.4% |
| Kibret et al (2014) | Amhara | IBCS | April 15 to May 14, 2012 | Pregnant women | 413 | 100% | 32.9% |
| Liyew et al (2017) | Amhara | IBCS | NR | Pregnant women | 285 | 100% | 28.4% |
| Melese et al (2016) | Amhara | CBCS | NR | Pregnant women | 690 | NR | 23.5% |
| Mohammed et al (2016) | Oromiya | IBCS | January 10 to April 13, 2015 | Pregnant women | 413 | 97.9% | 27.1% |
| Mulat et al (2017) | SNNPR | IBCS | NR | Pregnant women | 362 | 100% | 33.7% |
| Tebekaw et al (2014) | National level | EDHS | EDHS, 2011 | Women who had at least one birth | 7,759 | NA | 32% |
| Teshome et al (2010) | Amhara | CBCS | NR | currently married women | 576 | NR | 40.8% |
| Tsegaye et al (2018) | Amhara | CBCS | August to September | Married pregnant | 619 | 95.6% | 13.7% |

| | | | | | | | |
|---|---|---|---|---|---|---|---|
| | | | 2015 | women | | | |
| Wado et al (2013) | Oromiya | HDSS | March,2012 | mothers with alive birth in the two years | 1456 | 94% | 35% |
| Worku et al (2006) | Harar | CBCS | November to December 2001 | Reproductive age women | 983 | 98.3% | 33.3% |
| Yenealem et al (2019) | Amhara | CBCS | April 1–May 30, 2014 | Pregnant women | 325 | 100% | 20.6% |

*CBCS= Community-Based Cross Sectional Study, EDHS= Ethiopia Demographic and Health Survey, HDSS=Health and Demographic Surveillance System, IBCS=Institution-Based Cross Sectional Study, KDS-HRC= Kersa Demographic Surveillance and Health Research Center, NA= Not Applicable, NR=Not Report, SNNRP= Southern Nations, Nationalities, and Peoples' Region*

**Risk of bias assessment for the included studies**

We assessed the risk of bias for each of the original studies using the existing risk-of-bias assessment tool (**Table S1**). Of the total included studies, our summary assessment showed that more-than half (55.6%) of the studies had a low risk of bias, and nearly one-fourth (25.9%) of studies had a moderate risk of bias. Additionally, less than one-fifth (18.5%) of the included studies had a high risk of bias.

**Prevalence of unintended pregnancy in Ethiopia**

In this study, the overall prevalence of unintended pregnancy in Ethiopia was 28% (95% CI: 25-31) (**Fig 2**). We had conducted subgroup analyses to investigate how the prevalence of unintended pregnancy varies across different subgroups of studies (**Table 2**). Consequently, the pooled prevalence of unintended pregnancy was 26.4% (20.8-32.4) and 30.0% (26.6-33.6) for community-based cross-sectional and institution-based cross-sectional studies, respectively. The subgroup analysis based on the region where studies were conducted also showed that the highest prevalence of unintended pregnancy was observed from the Oromiya region [33.8%

(29.0-38.7)] followed by SNNPR [30.6% (25.2-36.2)] and Addis Ababa [28.0% (14.0-44.7)]. The lowest prevalence was also noted in Harar [22.9% (20.3-25.5)]. Furthermore, the pooled prevalence of unintended pregnancy was 28.2% (25.1-31.4) and 28.8% (23.7-34.0) for studies conducted prior and post 2014, respectively.

**Fig 2**. Forest plot of the pooled prevalence of unintended pregnancy in Ethiopia

**Table 2:** Subgroup analysis of studies included in meta-analysis on prevalence and determinants of unintended pregnancy in Ethiopia

| Subgroup | Random effects (95%CI) | Test of heterogeneity ($I^2$) |
|---|---|---|
| **By study design** | | |
| CBCS | 26.4% ( 20.8-32.4) | 96.6% |
| IBCS | 30.0% (26.6-33.6) | 84.4% |
| Overall | 28.2% (24.1-32.5) | 82.5% |
| **By region** | | |
| Amhara | 24.9% (19.2-31.0) | 95.2% |
| Oromiya | 33.8% (29.0-38.7) | 91.2% |
| SNNPR | 30.6% (25.2-36.2) | 87.6% |
| Addis Ababa | 28.0% (14.0-44.7) | 96.6% |
| National level | 28.0% (20.5-36.1) | 97.1% |
| Tigray | 26.5% (23.1-30.0) | - |
| Harar | 22.9% (20.3-25.5) | - |
| Overall | 26.5%(24.8-28.2) | 88.8% |
| **By data collection period** | | |
| 2014 and before | 28.2% (25.1-31.4) | 94.6% |
| After 2014 | 28.8% (23.7-34.0) | 94.5% |
| Overall | 28.4% (25.7-31.1) | 87.2% |

We assessed the issue of publication bias by visual inspection of funnel plot and by using the Egger's regression test. Though the funnel plot looks asymmetrical (**Fig 3**), the Egger's test

showed that no relationship between the effect size and its precision (P-value = 0.776). This might be due to heterogeneity in true effects.

**Fig 3**: Funnel plot of the prevalence of unintended pregnancy in Ethiopia.

**Determinants of unintended pregnancy in Ethiopia**

The significant determinants of unintended pregnancy reported from each study are presented in (**Table S2**).

In six studies, researchers examined the association between spousal communication about family planning and unintended pregnancy. Of this, five studies (26,30,36,37,41) showed that spousal communication about family planning was associated with an unintended pregnancy. The result of this meta-analysis showed that not communicating with one's husband about family planning was more likely to lead to unintended pregnancy (OR: 3.56, 95%CI: 1.68-7.53) (**Fig 4**). Ten studies were examined the association between the use of modern family planning methods and unintended pregnancy(10,22,26,29,34,37–39,41,42). Consequently, the result of this study showed that unintended pregnancy is more likely among women who never used family planning methods (OR: 2.08, 95%CI: 1.18-3.69) (**Fig.5**).

Of 21 studies that examined the association of maternal education level with unintended pregnancy, ten studies reported that maternal education status was associated with an unintended pregnancy (29,31,32,34–36,38–40). Furthermore, among the included studies, women's age, time to reach the nearest health facility, household wealth, and marital status showed significant association with unintended pregnancy.

**Fig 4**. The pooled odds ratio of the association between spousal communication about family planning and unintended pregnancy in Ethiopia.

**Fig 5**. The pooled odds ratio of the association between the use of family planning and unintended pregnancy in Ethiopia.

## Discussion

Unintended pregnancy has significant consequences for the health and welfare of women and children (7). Pregnancy intention in developing countries is influenced by socio-cultural, environmental, individual and health service-related factors (43). This systematic review and meta-analysis was conducted to determine the overall prevalence of unintended pregnancy and to identify its determinants in Ethiopia**.**

We found that the overall prevalence of unintended pregnancy in Ethiopia was 28% (95% CI: 25-31). This finding is consistent with the 2016 EDHS result (4), which reported that from all births in the past 5 years and current pregnancies, one-fourth (25%) were unintended. Additionally, in the previous two consecutive EDHS reports, we noted that the proportion of women who want no more births declined from 42% in 2005 to 37% in 2011. The prevalence of unintended pregnancy found in this study agreed with other previous studies conducted in Egypt (44), Ghana (45), and Bangladesh (9), which reported that nearly one-third (30.7%), 29.8% and 29% of pregnancies were unintended, respectively. However, the magnitude of unintended pregnancy found in this study was lower than previous studies conducted in Malawi, South Africa, and the Republic of Congo (46–49). This variation might be attributed to methodological differences in the assessment of pregnancy intention. The other possible reason for inconsistency in the prevalence of unintended pregnancy could be explained by the difference in the socio-economic characteristics of study participants (50–52).

In this study, we found that the pooled prevalence of unintended pregnancy was 26.4% and 30% for community-based cross-sectional and institutional-based cross-sectional studies, respectively. This variation could be explained by institution-based studies that include women who visit health facilities for legal abortion services, which is probably unintended pregnancy. About 97 % of women seeking an abortion reported having an unintended pregnancy (50). The highest prevalence of unintended pregnancy was observed from the Oromiya region (33.8%), and the lowest was in Harar (22.9%). The possible explanation might be due to the difference in the utilization of family planning methods.

In this systematic review and meta-analysis, we noted that a number of factors were found to be associated with an unintended pregnancy. Consequently, we found that women who hadn't communicated with their husbands about family planning methods were more likely to have an unintended pregnancy. The possible explanation for this result might be women's perception that their husbands oppose family planning, which is one of the dominant factors for discouraging the contraceptive practice in a wide variety of settings (20). Partner awareness of contraceptive and open discussion about family planning methods will decrease the risk of unintended pregnancy. The result of this study also showed that unintended pregnancy is more likely among women who never used family planning methods. This is due to the fact that awareness and proper utilization of modern contraceptives are crucial to reduce unintended pregnancy.

In this systematic review, unintended pregnancy was less likely among married women. The possible reason for this result might be that, in Ethiopian culture, pregnancy without marriage is unacceptable in most communities. This result is supported by previous studies conducted in South Africa and Kenya (53,54).

Though previous evidence stated that youngest women experience the highest rate of unintended pregnancy (55), in this systematic review, we observed controversial results regarding the association between women's age and unintended pregnancy. Some of the studies reported that younger women are more likely to have an unintended pregnancy, while other studies showed that older women are more likely to have unintended pregnancy.

Due to the inconsistent classification of maternal education level, time-lapse to reach the nearest health facility, parity, occupation and religion, we couldn't pool quantitatively, in this systematic review and meta-analysis. Contradictory results are observed between individual studies. Four studies (29,32,36,40) reported that women who have relatively better education were less likely to have an unintended pregnancy as compared to those who didn't have formal education. It is reasonable that as educational level increases the awareness of reproductive health also increases. Additionally, as the educational level increases, the spousal communication on pregnancy, awareness of long-term family planning, and receiving adequate ANC also increases. However, two studies reported that women who had relatively better education were more likely to have unintended pregnancy (31,39).

Moreover, in this study, we observed that as the time-lapse to reach the nearest health facility for providing contraceptives increased, women were more likely to have unintended pregnancy (10,29,42). It is also reasonable that as the time-lapse to reach the nearest health facility increases, the probability of missing ANC visits also increase, which in turn is associated with unintended pregnancy (13,20).

In this study, we noted that the parity of respondents showed a significant relationship with an unintended pregnancy. Consequently, multiparous women were more likely to experience an unintended pregnancy than nulliparous (34,35,40,56). This might be because of women who have attained their desired number of children will perceive any additional child as unwanted (57). Furthermore, the pregnancy of unemployed women is more likely to be unintended than employed women's (29,37).

Moreover, in this study, another important variable which significantly associated with unintended pregnancy was religion. Muslim mothers were less likely to report having an unintended pregnancy as compared to orthodox mothers. This finding is consistent with a study conducted in Ghana (58). This might be as a result of doctrinal differences among the women of along with different religions.

## Limitations

This study was not without limitations. Firstly, the review was limited to only articles published in the English language. Secondly, all of the included studies were cross-sectional, which limits assessment of the cause-effect relationships. Thirdly, we were unable to show the pooled odds ratio for all variables associated with unintended pregnancy because the included studies classified the variables in different ways.

## Conclusions

In this study, a high prevalence of unintended pregnancy was observed. Lack of spousal communication, never using family planning, maternal education, and household wealth level were significantly associated with an unintended pregnancy. This study implies the need to develop plans and policies to improve the awareness of contraceptive utilization and strengthen spousal communication related to pregnancy. Emphasis should be given to those women at a distant from health facilities, unmarried and teenagers.

## Refernces


1. Santelli BJ, Rochat R, Hatfield- K, Gilbert C, Curtis K, Cabral R, et al. The Measurement and Meaning of Unintended Pregnancy. 1999;94–101.
2. Unintended Pregnancy Prevention | Unintended Pregnancy - CDC-(Dec 23, 2016 - https://www.cdc.gov/reproductivehealth/contraception/unintendedpregnancy/index.htm.
3. Bearak J, Popinchalk A, Alkema L, Sedgh G. Global , regional , and subregional trends in unintended pregnancy and its outcomes from 1990 to 2014 : estimates from a Bayesian hierarchical model. Lancet Glob Heal [Internet]. 2014;6(4):e380–9. Available from: http://dx.doi.org/10.1016/S2214-109X(18)30029-9
4. Central Statistical Agency (CSA) [Ethiopia] and ICF. 2016. Ethiopia Demographic and Health Survey 2016. Addis Ababa, Ethiopia, and Rockville, Maryland, USA: CSA and

ICF.

5. Singh A, Singh A, Thapa S. Adverse Consequences of Unintended Pregnancy for Maternal and Child Health in Nepal. 2015;

6. Kabir, Sandra M.: Causes and Consequences of Unwanted Pregnancy from Asian Women's Perspectives. International Journal of Obstetrics. 1989. Supple 3 .p.9-14.

7. Mumah, J, Kabiru, CW, Mukiira, C, Brinton, J, Mutua, M, Izugbara, C, Birungi, H. and Askew, I. 2014. "Unintended Pregnancies in Kenya: A Country Profile," STEP UP Research Report. Nairobi: African Population and Health Research Center.

8. Induced Abortion and Postabortion Care in Ethiopia,Ipas,January 2017.

9. Rahman M, Rahman M, Tareque I, Ferdos J. Maternal Pregnancy Intention and Professional Antenatal Care Utilization in Bangladesh : A Nationwide Population-Based Survey. 2016;1–15.

10. Melese KG, Gebrie MH, Badi MB, Mersha WF. Unintended Pregnancy in Ethiopia : Community Based Cross-Sectional Study. 2016;2016.

11. Basinga P, Moore AM, Singh S, Remez L, Birungi F, Nyirazinyoye L. Unintended Pregnancy And Induced Abortion In Rwanda:Causes and Consequences. Guttmacher Inst.

12. Wado YD, Afework MF, Hindin MJ. Effects of Maternal Pregnancy Intention , Depressive Symptoms and Social Support on Risk of Low Birth Weight : A Prospective Study from Southwestern Ethiopia. 2014;9(5):1–7.

13. Wado YD, Afework MF, Hindin MJ. Unintended pregnancies and the use of maternal health services in southwestern Ethiopia. BMC Int Health Hum Rights [Internet]. 2013;13(1):1. Available from: BMC International Health and Human Rights

14. Cochrane Book Series. Cochrane Handbook for Systematic Reviews of Interventions.

15. Hoy D, Brooks P, Woolf A, Blyth F, March L, Bain C, et al. Assessing risk of bias in prevalence studies : modification of an existing tool and evidence of interrater agreement. J Clin Epidemiol. 2012;65(9):934–9.

16. Wang N. How to Conduct a Meta-Analysis of Proportions in R : A Comprehensive Tutorial Conducting Meta-Analyses of Proportions in R. 2018;(June).

17. Higgins JPT, Thompson SG, Deeks JJ, Altman DG. Measuring inconsistency in meta-analyses. :557–60.

18. Abame DE, Abera M, Tesfay A, Yohannes Y, Ermias D, Markos T, et al. Relationship

Between Unintended Pregnancy and Antenatal Care Use During Pregnancy in Hadiya Zone, Southern Ethiopia. J Reprod Infertil. 2019;20(1):42–51.
19. Worku S, Fantahun M. Unintended pregnancy and induced abortion in a town with accessible family planning services : The case of Harar in eastern Ethiopia.
20. Sectional C, Abayu H, Birhanu Z, Nega A, Kidanemariam A. Prevalence and Associated Factors of Unintended Pregnancy in Welkaite. 2015;2(2).
21. Admasu E, Mekonnen A, Setegn T, Abeje G. Level of unintended pregnancy among reproductive age women in Bahir Dar city administration , Northwest Ethiopia. BMC Res Notes. 2018;1–5.
22. Fite RO, Mohammedamin A, Abebe TW. Unintended pregnancy and associated factors among pregnant women in Arsi Negele. BMC Res Notes. 2018;1–7.
23. Gite A, Liulseged N, Seyife H, Abrha Y, Workineh Y, Shegaze M, et al. Unintended Pregnancy: Magnitude and Associated Factors among Pregnant Women in Arba Minch Town, Gamo Gofa Zone, Ethiopia, 2015. Reprod Syst Sex Disord Curr Res. 2016;5(4):6.
24. Hamdela B, Abebe G, Tilahun T. Unwanted Pregnancy and Associated Factors among Pregnant Married Women in Hosanna Town , Southern. 2012;7(6).
25. Teshome FT, Hailu AG, Teklehaymanot AN. Prevalence of unintended pregnancy and associated factors among married pregnant women in Ganji woreda , west Wollega Oromia region , Ethiopia. 2014;2(2):92–101.
26. Tsegaye AT, Mengistu M, Shimeka A. Prevalence of unintended pregnancy and associated factors among married women in west Belessa Woreda , Northwest. 2018;1–8.
27. Yenealem F, Niberet G. Prevalence and associated factors of unintended pregnancy among pregnant woman in Gondar town , North west Ethiopia ,. BMC Res Notes. 2019;1–5.
28. Health H, Shewa N. Assessment of the Prevalence of Unplanned Pregnancy and Associated Factors among Pregnant Women Attending Antenatal Care Unit at Journal of Women ' s Health Care. 2017;6(6).
29. Darega B, Dida N. Unplanned Pregnancy: Prevalence and Associated Factors among Antenatal Care Attending Women in Bale Zone, Oromiya Region, Southeast Ethiopia: A Facility - based Cross Sectional Study. 2015;15(4).
30. Liyew B, Desalegn B, Ayalew Y. Prevalence of Unintended Pregnancy and Associated

Factors among Currently Pregnant Mothers in Kombolcha, Southern Wollo, Ethiopia, 2017.

31. Feyisso M, Girma A, Yimam H, Hailu S. Journal of Women ' s Health Care Magnitude of Unintended Pregnancy and its Determinants among Pregnant Women Visiting Dilla University Referral Hospital , South. 2017;6(4).

32. Gebreamlak W, Aragaw A, Lemma S, Demilew W. Magnitude and factors influencing unintended pregnancy among pregnant women attending antenatal care at Felege Hiwot referral hospital , Northwest Ethiopia : A cross-sectional study. 2014;2(4):261–9.

33. Gizaw W, Fetene G, Mohammed S, Alemu A. iMedPub Journals Magnitude of Unplanned Pregnancy and its Associated Factors among Pregnant Women Attending Antenatal Care at Tepi General Hospital Sheka. 2018;1–8.

34. Goshu YA, Yitayew AE (2019) Prevalence and determinant factors of unintended pregnancy among pregnant women attending antenatal clinics of Addis Zemen hospital. PLoS ONE 14(1): e0210206. https://doi.org/10.1371/journal. pone.0210206.

35. Kahasay S, Shemelis T. Assesment of magnitude and factors associated with unintended pregnancy among preparatory highschool students,Addis Ababa,Ethiopia. 2015

36. Kassie T, Geteneh M, Ali A, Tefera W. Magnitude and factors associated with unintended pregnancy among pregnant women in Addis Ababa, Ethiopia. Glob J Med PUBLIC Heal. 2017;6(4):15.

37. Kibret A, Bayu H, Merga M. Prevalence of Unintended Pregnancy and Associated Factors among Pregnant Women Attending Antenatal Clinics in Debre-markos Town, North West Ethiopia 2012. Women's Heal Care. 2014;4(3).

38. Mulat S, Fekadu M, Abera H, Bekele G, Bedaso A. Journal of Women ' s Health Care Prevalence of Unplanned Pregnancy and Associated Factors among Mothers Attending Antenatal Care at Hawassa City Public Hospitals , Hawassa , SNNPR ,. 2017;6(4).

39. Habte D, Teklu S, Melese T, Magafu MGMD. Correlates of Unintended Pregnancy in Ethiopia : Results From a National Survey. 2013;8(12):1–8.

40. Tebekaw Y, Aemro B, Teller C. Prevalence and determinants of unintended childbirth in Ethiopia. 2014;1–9.

41. Abayu H, Birhanu Z, Nega A, Kidanemariam3 A. Prevalence and Associated Factors of Unintended Pregnancy in Welkaite Woreda, Tigray and North Ethiopia Cross Sectional

Study by 2012. J Pregnancy Child Heal. 2015;2(2).
42. Kassa N, Berhane Y, Worku A. Predictors of unintended pregnancy in Kersa ,. 2012;1–7.
43. Yakubu I, Salisu WJ. Determinants of adolescent pregnancy in sub-Saharan Africa: a systematic review. BMC Reprod Heal. 2018;
44. Mohamed EAB, Hamed AF, Yousef FMA, Ahmed EA. Prevalence , determinants , and outcomes of unintended pregnancy in Sohag district , Egypt. 2019;9.
45. Ameyaw EK. Prevalence and correlates of unintended pregnancy in Ghana: Analysis of 2014 Ghana Demographic and Health Survey. Matern Heal Neonatol Perinatol. 2018;4(1):1–6.
46. Palamuleni ME, Adebowale AS. Prevalence and determinants of unintended pregnancies in Malawi. African Population Studies. 2014 Apr 29;28(1):551-63.
47. Adeniyi OV, Ajayi AI, Moyaki MG, Goon D Ter, Avramovic G, Lambert J. High rate of unplanned pregnancy in the context of integrated family planning and HIV care services in South Africa. BMC Health Serv Res. 2018;18(1):4–11.
48. Hall JA, Barrett G, Phiri T, Copas A, Malata A, Stephenson J (2016) Prevalence and Determinants of Unintended Pregnancy in Mchinji District, Malawi; Using a Conceptual Hierarchy to Inform Analysis. PLoS ONE 11(10): e0165621. doi:10.1371/journal.pone.01656.
49. Ndziessi G, Bitemo M, Kaboru BB. Unintended Pregnancies and Associated Factors among Contraceptive Users: a Study from Referral Hospitals in Brazzaville, Republic of Congo. SM J Community Med. 2016;2(1):1015.
50. Metcalfe A, Talavlikar R, Du Prey B, Tough SC. Exploring the relationship between socioeconomic factors, method of contraception and unintended pregnancy. Reprod Health [Internet]. 2016;13(1):1–8. Available from: http://dx.doi.org/10.1186/s12978-016-0151-y
51. Omani-Samani R, Amini Rarani M, Sepidarkish M, Khedmati Morasae E, Maroufizadeh S, Almasi-Hashiani A. Socioeconomic inequality of unintended pregnancy in the Iranian population: A decomposition approach. BMC Public Health. 2018;18(1):1–8.
52. Roy TK, Singh BP (2016). Correlates of Unwanted Births in Bangladesh: A Study through Path Analysis. PLoS ONE 11(10): e0164007. https://doi.org/10.1371/journal.pone.0164007.

53. Africa S, Haffejee F, Connor LO, Govender N, Reddy P, Sibiya MN, et al. Factors associated with unintended pregnancy among women attending a public health facility in Factors associated with unintended pregnancy among women attending a public health facility in KwaZulu-Natal , South Africa. South African Fam Pract [Internet]. 2018;6190:1–5. Available from: http://doi.org/10.1080/20786190.2017.1396790
54. Ikamari L, Izugbara C, Ochako R. Prevalence and determinants of unintended pregnancy among women in Nairobi , Kenya. 2013;1–9.
55. Finer LB et al. Contraception. 2011;84:478–485.
56. Mohammed F, Musa A, Amano A. Prevalence and determinants of unintended pregnancy among pregnant woman attending ANC at Gelemso General Hospital , Oromiya Region , East Ethiopia : a facility based cross-sectional study. BMC Womens Health [Internet]. 2016;10–6. Available from: http://dx.doi.org/10.1186/s12905-016-0335-1
57. Addis K, Bayu H, Merga M. Prevalence of Unintended Pregnancy and Associated Factors among Pregnant Women Attending Antenatal Clinics in Debre-markos Town, North West Ethiopia 2012. Women's Heal Care. 2014;4(3):6.
58. Ameyaw EK. Prevalence and correlates of unintended pregnancy in Ghana: Analysis of 2014 Ghana Demographic and Health Survey. Maternal health, neonatology and perinatology. 2018 Dec;4(1):17.

**Table S1**: Assessing the risk of bias for the included studies

**Table S2**: Significant determinants of unintended pregnancy reported from each study.

**Author's response to reviews**

**Title**: **Prevalence and determinants of unintended pregnancy in Ethiopia: a systematic review and meta-analysis of observational studies**

**Authors**

**Author's email addresses**

MA: mulunehadis@gmail.com

LY: lielt.yismaw@gmail.com

YB: yebelay.ma@gmail.com

BK: bekalukassiedmu@gmail.com

RY: reta.yeshambel@gmail.com

MAA: agazhemoges@gmail.com

**Date: 17 October 2019**

**Dear Editor,**

We thank you for the chance to resubmit our revised manuscript. Also, we would like to thank the reviewers for sharing the view and experience. The comments are very important that will improve the manuscript. The point-by-point responses for each of the comments are provided in the following pages. We hope that the revisions meet your standards and that the paper would be published in your journal. We look forward to working with you towards a final published product.

Sincerely,

Muluneh Alene, MPH

On behalf of co-authors

**Point by point responses to queries**

**Reviewer#1**

| **Comments and points raised** | **Authors response** |
|---|---|
| 1. “Delete first sentence of abstract?” | **Answer: thank you dear reviewer for your constructive comments!**<br>○ Based on the suggestion given, we make modification on the first sentence of the abstract section in the revised form of the manuscript. |
| 2. “The quality of the English needs major work.” | **Answer: Thank you!**<br>○ The issue of English language is resolved using English language professionals in the revised form of the manuscript. |

**Reviewer#2**

| **Comments and points raised** | **Authors response** |
|---|---|
| 1. “Was a time period determined for the search? If yes, what was it? If no, then please mention that.” | **Answer: thank you dear reviewer for your constructive comments!**<br>○ The searching time period was already stated and it was between the 15$^{th}$ of February and 30$^{th}$ of April 2019 |
| 2. “How did you search for unpublished studies?” | **Answer: Thank you!**<br>○ To find unpublished papers relevant to this study, some research centers including Addis Ababa digital library were searched. |
| 3. “Was any attempt made to contact authors of studies for which full papers were not obtained?” | **Answer: Thank you!**<br>○ We were planned to contact the primary author by email at least three times if a study is not fully available. But, studies searched for this systematic review and |

| | |
|---|---|
| | meta-analysis were fully obtained. |
| 4. "The writing needs quite a bit of improvement in terms of flow." | **Answer: Thank you!**<br>○ Based on your suggestion, we revised the flow of idea in the revised form of the manuscript and we make highlight for changes. |

**Reviewer#3**

| **Comments and points raised** | **Authors response** |
|---|---|
| 1. "Literature search: it is limited to 2 data bases and Google scholar, which is not comprehensive. The authors should have searched more relevant databases. They mentioned that they searched national databases, however, there is no description of such database. The search was limited to English language. As this study is investigating the prevalence of unintended pregnancy in a specific county/ region, it must have been included the articles in local language. This may increase the risk of reporting and publication bias." | **Answer: thank you dear reviewer for your constructive comments!**<br>○ Based on the comments given and to make comprehensive, in the revised form of the manuscript, we extend our searches for studies from PubMed/MEDLINE, Web of Science, CINAHL, Google Scholar, Science Direct and Cochrane Library.<br>○ We were searched the "Ethiopian Journal of Public Health and Nutrition" for the national databases.<br>○ You are right dear reviewer; not including articles published in local language may increase the risk of reporting and publication bias.<br>○ But, we were searched for articles published in local language related to this study, but we can't get such articles. |
| 2. "Authors should explain the reasons in heterogeneity among studies. Why it exists, and how it affects the credibility | **Answer: Thank you!**<br>○ Based on the comment given, we included the possible reasons for |

| | |
|---|---|
| of the overall results?” | heterogeneity among included studies.<br>○ In this systematic review and meta-analysis, the possible sources of heterogeneity among studies are differences in study participants, study design, risk of bias and data collection period.<br>○ To account the heterogeneity among the included studies, subgroup analysis and a random effect meta-analysis with an estimation of DerSimonian and Laird method was performed. |
| 3. “Discussion: there is no mention of women’s age, time to reach the nearest health facility, household wealth, being married… in results section, but they show up in dissuasion as the potential determinants on the prevalence of unintended pregnancies” | ○ Based on the comments given, we included the stated variables in the result section. |

**Reviewer#4**

| Comments and points raised | Authors response |
|---|---|
| 1. “Parity, occupation, religion and economic status are important determinants of unintended pregnancy. Could the authors provide insight into why they were not significantly associated with unintended pregnancy in the review?” | **Answer: thank you dear reviewer for your constructive comments!**<br>○ In the revised form of the manuscript, we already considered the association between the mentioned variables and unintended pregenancy. |
| 2. “As readers may not be familiar with | **Answer: Thank you!** |

| | |
|---|---|
| Ethiopia, please define SNNPR at first instance.” | ○ Based on the comment given, we already define the word “SNNPR” at first instance in the revised form of the manuscript. |

**Reviewer#5**

| **Comments and points raised** | **Authors response** |
|---|---|
| 1. “There are 195 English language corrections that I strongly suggest you follow in the Comments section of the attached PDF file.” | **Answer: thank you dear reviewer for your constructive comments!**<br>○ We thank you for your revision, and we considered all English language corrections in the revised form of the manuscript. |

**Thank you!!!**
