# Supplementary material for: Prevalence and determinants of unintended pregnancy in Ethiopia: A systematic review and meta-analysis of observational studies
Source: PLoS One. 2020 Apr 7;15(4):e0231012. doi: 10.1371/journal.pone.0231012 (PMC7138300; doi:10.1371/journal.pone.0231012)
Supplement: Attachment — Submitted filename: Response to Reviewers.docx [file pone.0231012.s006.docx]

**Author's response to reviews**

**Title**: **Prevalence and determinants of unintended pregnancy in Ethiopia: a** **systematic review and meta-analysis of observational studies**

**Authors**

**Author’s email addresses**

MA: [mulunehadis@gmail.com](mailto:mulunehadis@gmail.com)

LY: [lielt.yismaw@gmail.com](mailto:lielt.yismaw@gmail.com)

YB: [yebelay.ma@gmail.com](mailto:yebelay.ma@gmail.com)

BK: [bekalukassiedmu@gmail.com](mailto:bekalukassiedmu@gmail.com)

RY: [reta.yeshambel@gmail.com](mailto:reta.yeshambel@gmail.com)

MAA: [agazhemoges@gmail.com](mailto:agazhemoges@gmail.com)

**Date: 17 October 2019**

**Dear Editor,**

We thank you for the chance to resubmit our revised manuscript. Also, we would like to thank the reviewers for sharing the view and experience. The comments are very important that will improve the manuscript. The point-by-point responses for each of the comments are provided in the following pages. We hope that the revisions meet your standards and that the paper would be published in your journal. We look forward to working with you towards a final published product.

Sincerely,

Muluneh Alene, MPH

On behalf of co-authors

**Point by point responses to queries**

**Reviewer#1**

| **Comments and points raised** | **Authors response** |
| --- | --- |
| 1. “Delete first sentence of abstract?” | **Answer: thank you dear reviewer for your constructive comments!**   - Based on the suggestion given, we make modification on the first sentence of the abstract section in the revised form of the manuscript. |
| 2. “The quality of the English needs major work.” | **Answer: Thank you!**   - The issue of English language is resolved using English language professionals in the revised form of the manuscript. |

**Reviewer#2**

| **Comments and points raised** | **Authors response** |
| --- | --- |
| 1. “Was a time period determined for the search? If yes, what was it? If no, then please mention that.” | **Answer: thank you dear reviewer for your constructive comments!**   - The searching time period was already stated and it was between the 15^th^ of February and 30^th^ of April 2019 |
| 2. “How did you search for unpublished studies?” | **Answer: Thank you!**   - To find unpublished papers relevant to this study, some research centers including Addis Ababa digital library were searched. |
| 3. “Was any attempt made to contact authors of studies for which full papers were not obtained?” | **Answer: Thank you!**   - We were planned to contact the primary author by email at least three times if a study is not fully available. But, studies searched for this systematic review and meta-analysis were fully obtained. |
| 4. “The writing needs quite a bit of improvement in terms of flow.” | **Answer: Thank you!**   - Based on your suggestion, we revised the flow of idea in the revised form of the manuscript and we make highlight for changes. |

**Reviewer#3**

| **Comments and points raised** | **Authors response** |
| --- | --- |
| 1. “Literature search: it is limited to 2 data bases and Google scholar, which is not comprehensive. The authors should have searched more relevant databases. They mentioned that they searched national databases, however, there is no description of such database. The search was limited to English language. As this study is investigating the prevalence of unintended pregnancy in a specific county/ region, it must have been included the articles in local language. This may increase the risk of reporting and publication bias.” | **Answer: thank you dear reviewer for your constructive comments!**   - Based on the comments given and to make comprehensive, in the revised form of the manuscript, we extend our searches for studies from PubMed/MEDLINE, Web of Science, CINAHL, Google Scholar, Science Direct and Cochrane Library. - We were searched the “Ethiopian Journal of Public Health and Nutrition” for the national databases. - You are right dear reviewer; not including articles published in local language may increase the risk of reporting and publication bias. - But, we were searched for articles published in local language related to this study, but we can’t get such articles. |
| 2. “Authors should explain the reasons in heterogeneity among studies. Why it exists, and how it affects the credibility of the overall results?” | **Answer: Thank you!**   - Based on the comment given, we included the possible reasons for heterogeneity among included studies. - In this systematic review and meta-analysis, the possible sources of heterogeneity among studies are differences in study participants, study design, risk of bias and data collection period. - To account the heterogeneity among the included studies, subgroup analysis and a random effect meta-analysis with an estimation of DerSimonian and Laird method was performed. |
| 3. “Discussion: there is no mention of women’s age, time to reach the nearest health facility, household wealth, being married… in results section, but they show up in dissuasion as the potential determinants on the prevalence of unintended pregnancies” | - Based on the comments given, we included the stated variables in the result section. |

**Reviewer#4**

| **Comments and points raised** | **Authors response** |
| --- | --- |
| 1. “Parity, occupation, religion and economic status are important determinants of unintended pregnancy. Could the authors provide insight into why they were not significantly associated with unintended pregnancy in the review?” | **Answer: thank you dear reviewer for your constructive comments!**   - In the revised form of the manuscript, we already considered the association between the mentioned variables and unintended pregenancy. |
| 2. “As readers may not be familiar with Ethiopia, please define SNNPR at first instance.” | **Answer: Thank you!**   - Based on the comment given, we already define the word “SNNPR” at first instance in the revised form of the manuscript. |

**Reviewer#5**

| **Comments and points raised** | **Authors response** |
| --- | --- |
| 1. “There are 195 English language corrections that I strongly suggest you follow in the Comments section of the attached PDF file.” | **Answer: thank you dear reviewer for your constructive comments!**   - We thank you for your revision, and we considered all English language corrections in the revised form of the manuscript. |

**Thank you!!!**
