# Supplementary material for: Prevalence and determinants of unintended pregnancy in Ethiopia: A systematic review and meta-analysis of observational studies
Source: PLoS One. 2020 Apr 7;15(4):e0231012. doi: 10.1371/journal.pone.0231012 (PMC7138300; doi:10.1371/journal.pone.0231012)
Supplement: Attachment — Submitted filename: Response to Reviewers .docx [file pone.0231012.s008.docx]

**Author's response to reviews**

**Title**: **Prevalence and determinants of unintended pregnancy in Ethiopia: a** **systematic review and meta-analysis of observational studies**

**Authors**

**Author’s email addresses**

MA: [mulunehadis@gmail.com](mailto:mulunehadis@gmail.com)

LY: [lielt.yismaw@gmail.com](mailto:lielt.yismaw@gmail.com)

YB: [yebelay.ma@gmail.com](mailto:yebelay.ma@gmail.com)

BK: [bekalukassiedmu@gmail.com](mailto:bekalukassiedmu@gmail.com)

RY: [reta.yeshambel@gmail.com](mailto:reta.yeshambel@gmail.com)

MAA: [agazhemoges@gmail.com](mailto:agazhemoges@gmail.com)

**Date: 27 November 2019**

**Dear Editor,**

We thank you for the chance to resubmit our revised manuscript. Also, we would like to thank the reviewers for sharing the view and experience. The comments are very important that will improve the manuscript. The point-by-point responses for each of the comments are provided in the following pages. We hope that the revisions meet your standards and that the paper would be published in your journal. We look forward to working with you towards a final published product.

Sincerely,

Muluneh Alene, MPH

On behalf of co-authors

**Point by point responses to queries**

**Reviewer#2**

| **Comments and points raised** | **Authors response** |
| --- | --- |
| The time period of the search is still not mentioned. Also in the flowchart, the further breakdown of how many studies were excluded because the full study was not available to them and how many did not have the outcome of interest is needed. What all efforts were done to try and get the full study? Were the authors contacted? | **Answer: thank you dear reviewer for your constructive comments!**   - The searching time period was already stated in the previous revised form of the manuscript: **Page 6, line 168-169.** - In **Fig. 1 (flow chart diagram)**, we included the number of studies excluded because the outcome of interest did not report, having data that were not extractable and conducted in other countries. - We were planned to contact the primary author by email at least three times if a study is not fully available. But, studies searched for this systematic review and meta-analysis were fully obtained. |

**Reviewer#5**

| **Comments and points raised** | **Authors response** |
| --- | --- |
| 1. “There are still 12 more minor corrections remaining.” | **Answer: thank you dear reviewer for your constructive comments!**   - We thank you again for your revision, and we considered all English language corrections in the revised form of the manuscript. - In addition, we used English language professionals to write the manuscript in Standard English. |
