# Supplementary material for: Prevalence and determinants of unintended pregnancy in Ethiopia: A systematic review and meta-analysis of observational studies
Source: PLoS One. 2020 Apr 7;15(4):e0231012. doi: 10.1371/journal.pone.0231012 (PMC7138300; doi:10.1371/journal.pone.0231012)
Supplement: Attachment — Submitted filename: Response to Reviewers.docx [file pone.0231012.s009.docx]

**Author's response to reviews**

**Title**: **Prevalence and determinants of unintended pregnancy in Ethiopia: a** **systematic review and meta-analysis of observational studies**

**Authors**

**Author’s email addresses**

MA: [mulunehadis@gmail.com](mailto:mulunehadis@gmail.com)

LY: [lielt.yismaw@gmail.com](mailto:lielt.yismaw@gmail.com)

YB: [yebelay.ma@gmail.com](mailto:yebelay.ma@gmail.com)

BK: [bekalukassiedmu@gmail.com](mailto:bekalukassiedmu@gmail.com)

RY: [reta.yeshambel@gmail.com](mailto:reta.yeshambel@gmail.com)

MAA: [agazhemoges@gmail.com](mailto:agazhemoges@gmail.com)

**Date: 25 January 2020**

**Dear Editor,**

We thank you for the chance to resubmit our revised manuscript. Also, we would like to thank the reviewers for sharing the view and experience. The comments are very important that will improve the manuscript. The point-by-point responses for each of the comments are provided in the following pages. We hope that the revisions meet your standards and that the paper would be published in your journal. We look forward to working with you towards a final published product.

Sincerely,

Muluneh Alene, MPH

On behalf of co-authors

**Point by point responses to queries**

**Reviewer#2**

| **Comments and points raised** | **Authors response** |
| --- | --- |
| 1. Line73"...25% were unintended...", Line 82 it is not 100% that a woman WILL have low mental and physical health, I would reword it and say that women with unintended pregnancy are more likely to have low mental and physical health | **Answer: thank you dear reviewer for your constructive comments!**   - In the revised form of the manuscript, we already incorporate your suggestion. |
| 2. My question about the time period of articles that were searched still remains unclear, the time period mentioned 15 February to 30 April 2019 is the time period during which the search was conducted or only articles published within this time period was included? Because if it is the latter, which I doubt it is, then that is a very short time period. This needs to be clearly mentioned in the article. | **Answer: thank you dear reviewer for your constructive comments which are very important to improve the manuscript!**   - The time period what we mentioned in **page 6, Line 169-170** (15 February to 30 April 2019) is only the period what we search for studies. - In this study, we included all existing articles without considering the year of publication. So, as it indicates in **page 6, Line 177-178** the publication year among the included studies was between 2006 and 2019. - We make highlight for changes in the revised form of the manuscript. |

**Thank you!!!**
